# Taming Electrowetting Using Highly Concentrated Aqueous Solutions

**DOI:** 10.1021/acs.jpcc.2c06517

**Published:** 2022-11-30

**Authors:** Athanasios A. Papaderakis, Kacper Polus, Pallav Kant, Finn Box, Bruno Etcheverry, Conor Byrne, Martin Quinn, Alex Walton, Anne Juel, Robert A. W. Dryfe

**Affiliations:** † Department of Chemistry, University of Manchester, Oxford Road, ManchesterM13 9PL, United Kingdom; ‡ Henry Royce Institute, University of Manchester, Oxford Road, ManchesterM13 9PL, United Kingdom; § Photon Science Institute, University of Manchester, Oxford Road, ManchesterM13 9PL, United Kingdom; ∥ Department of Physics and Astronomy, Manchester Center for Nonlinear Dynamics, University of Manchester, Oxford Road, ManchesterM13 9PL, United Kingdom

## Abstract

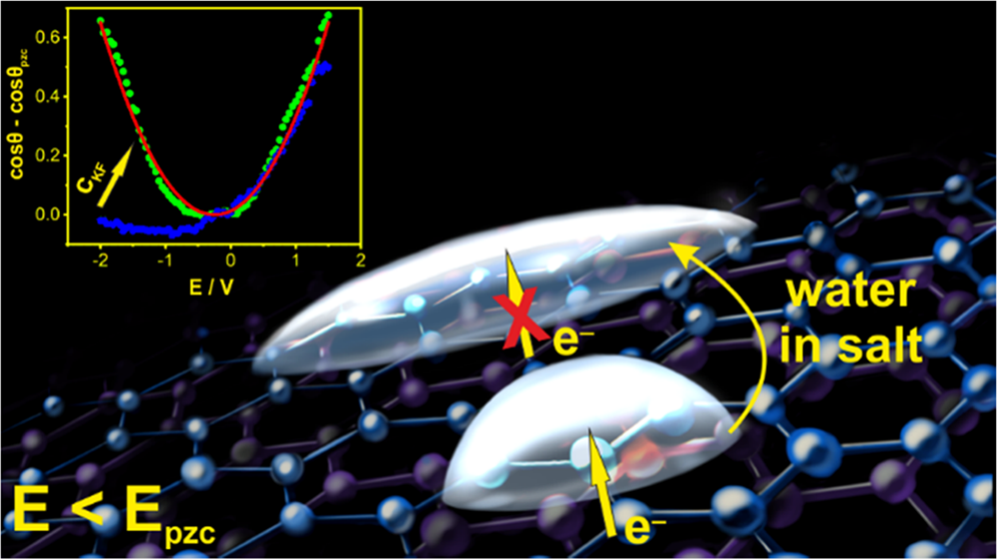

Wetting of carbon surfaces is one of the most widespread, yet poorly understood, physical phenomena. Control over wetting properties underpins the operation of aqueous energy-storage devices and carbon-based filtration systems. Electrowetting, the variation in the contact angle with an applied potential, is the most straightforward way of introducing control over wetting. Here, we study electrowetting directly on graphitic surfaces with the use of aqueous electrolytes to show that reversible control of wetting can be achieved and quantitatively understood using models of the interfacial capacitance. We manifest that the use of highly concentrated aqueous electrolytes induces a fully symmetric and reversible wetting behavior without degradation of the substrate within the unprecedented potential window of 2.8 V. We demonstrate where the classical “Young–Lippmann” models apply, and break down, and discuss reasons for the latter, establishing relations among the applied bias, the electrolyte concentration, and the resultant contact angle. The approach is extended to electrowetting at the liquid|liquid interface, where a concentrated aqueous electrolyte drives reversibly the electrowetting response of an insulating organic phase with a significantly decreased potential threshold. In summary, this study highlights the beneficial effect of highly concentrated aqueous electrolytes on the electrowettability of carbon surfaces, being directly related to the performance of carbon-based aqueous energy-storage systems and electronic and microfluidic devices.

## 1 Introduction

Nature’s subtle control of small amounts of liquids in various physicochemical processes has served as an inspiration to the artificial manipulation of wetting phenomena. These efforts aim to tackle fundamental challenges in physics,^1^ chemistry,^2^ and biology^3,4^ and pave the way toward the development of novel devices with a vast range of applications. The ability to actuate droplets of liquid using external signals,^5^ such as electricity^6,7^ or light irradiation,^8−10^ is utilized in several technological areas including optics,^11−14^ displays,^15,16^ and “lab-on-a-chip” systems.^17,18^ The reduction in the contact angle (CA) upon application of an external voltage at the solid|liquid interface is referred to as electrowetting.^6^ Despite significant advances in the state-of-the-art technology of electrowetting on dielectrics (EWODs),^17^ persistent reliability issues continue to emerge.^19^ Typical problems involve dielectric breakdown^20^ and charging^21^ as well as the high cost of the devices imposed by the advanced architecture and the preparation methods of the materials used.^19^

Moreover, electrowetting has started to attract further interest due to major recent developments in aqueous and nonaqueous energy-storage systems, such as electrochemical supercapacitors. The power and energy-storage performance of these devices are directly linked to the electrowettability of electrodes^22,23^ since only the surface area wetted by the electrolyte contributes to the overall capacitance. Therefore, characterization and control of electrowetting under diverse operating conditions are crucial for the manufacturing of effective and reliable energy-storage devices.

Herein, we investigate the electrowetting behavior of the basal plane of highly oriented pyrolytic graphite (HOPG) as a model carbon system in direct contact with aqueous solutions of alkali metal halides. We show that a decrease in the electrolyte concentration results in a highly asymmetric dependence of the apparent contact angle (CA), θ, on the applied potential, *E*, vs the potential of zero charge (*E*_pzc_). This phenomenon is interpreted in terms of electrochemically induced charge transfer processes occurring on graphite and their effects on the total charge of the interface. To effectively control these processes and boost the electrowetting response of graphite, we implement the newly introduced class of electrolytes referred to as “water-in-salt” electrolytes.^24−26^ In this way, the purely capacitive potential region of the interface—and hence the operational window of the system—can be extended up to 2.8 V, which is the highest stable operational window reported to date for electrowetting on conductors (EWOCs) liquid–air or liquid–liquid configuration. Within this potential window, CA changes of more than 45° are recorded. The approach is extended to the liquid|liquid interface, an important configuration from the optofluidic device development point of view, where changes in the potential-dependent substrate–electrolyte surface energy induce fully reversible CA changes in an insulating organic phase serving as the heavy phase.

## 2 Methods

### 2.1 Electrowetting Configuration

A sketch of the setup for liquid|air electrowetting can be seen in Figure S1a. A microinjector (PV820 Pneumatic PicoPump, from World Precision Instruments, FL) was used to expel the electrolyte solution from a micropipette and deposit the droplet on the HOPG. The former was fabricated by pulling a borosilicate capillary (inner diameter 0.84 mm, outer diameter 1.5 mm, length 10.16 cm, from World Precision Instruments, U.K.) with a Sutter P-97 Flaming/Brown micropipette puller. The inner diameter of the tip in the resulted micropipettes was ca. 5–6 μm. A platinum wire (99.99% purity, 0.05 mm diameter, from Advent, U.K.), carefully placed on the upper inner part of the micropipette so as not to touch the bare part of the AgCl wire (used as a reference electrode, RE), was employed as a counter electrode (CE). The position of the wires was secured by the rubber gasket between the capillary and the pipette holder. When necessary, to avoid evaporation of the electrolyte during the experiments, the HOPG sample was surrounded by glass cells filled with ultrapure water to maintain high humidity conditions in its immediate vicinity. The position of both the HOPG and the micropipette was controlled using manual micropositioners (Thor Labs). The micropipette was brought close to the surface of the working electrode, and the smoothest regions of the HOPG were targeted. A Photron FASTCAM SA3 high speed camera controlled via Photron FASTCAM Viewer and a Storz Xenon Nova 300 light source were used in static and dynamic experiments. In the case of the dynamic measurements, the frame rate (50 fps for the stability tests in Figures 6 and 8c,d and 6000 fps for the high-resolution data in Figure 7) was adjusted based on preliminary experiments to successfully probe the timescales of the droplet’s advancing/receding motions within the timeframe of each experiment (i.e., recording at least 20 points between the equilibrium CA plateaus for the wetting/dewetting states). The characteristic times for the advancing and receding motions used for the determination of timescales were taken to be 90% of the corresponding CA final values. For the liquid|liquid electrowetting, the setup is schematically displayed in Figure S1c. Overall, the experimental configuration is similar to the one used for the liquid|air experiments with the main differences being the use of a quartz container filled with the surrounding electrolyte, in which HOPG was immersed and the absence of the pipette. The latter was used solely for the deposition of the heavy phase on HOPG, and subsequently it was retracted. In this configuration, a Pt mesh (from Advent, U.K.) and a custom-made Ag/AgCl_(sat.KCl)_ electrode (see Section S1.2 in the SI) were used as the CE and RE, respectively.

### 2.2 Electrochemical Measurements

All electrochemical experiments were performed on an Autolab PGSTAT302N potentiostat from Metrohm equipped with the FRA32 module and operated with Nova 1.11.2 software. Each measurement was conducted on a freshly cleaved HOPG surface. Before the experiment, the CE was flame-cleaned with a blue butane flame and the RE was thoroughly washed with DI water. To avoid the contamination of the HOPG by the adsorption of air-bound hydrocarbons, a phenomenon well established in the literature for the basal plane of graphite,^27^ the solution was deposited on the WE within 1 min of cleaving the surface. Unless specified otherwise, the applied potential, *E*, throughout the main text is referred vs Ag/AgCl_(sat. KCl)_ (see Section S1.2 in the SI). The experimental protocol used for the static measurements was composed of consecutive potential pulses from 0 to −2 V vs Ag/AgCl wire with a step of 50 mV. For the positive side, the same approach was followed in the potential range between 0 and +1.5 V vs Ag/AgCl wire. The duration of the pulses was adjusted accordingly (varying from 5 to 15 s) for each electrolyte concentration in the range of 0.1–16 m for KF and 0.1–20 m for CsF based on preliminary dynamic experiments for the determination of the time required to attain equilibrium; the latter was indicated by the plateau in the calculated CA values. A similar strategy was adopted for the dynamic measurements, in which the potential was directly stepped from 0 to either −2 or +1.5 V vs Ag/AgCl wire three consecutive times. Each repetition represents one cycle. Once again, the duration of the potential pulse was chosen based on the required time needed (0.5 s) to ensure a steady state response for the electrolyte concentration used. For the investigation of the surface processes occurring during cathodic and anodic polarization in different electrolyte concentrations, cyclic voltammetry (CV) experiments were carried out over a potential range from 0 to −2 and 0 to +1.5 V vs Ag/AgCl_(sat. KCl)_, respectively, at a scan rate of 1 V s^–1^. Electrochemical impedance spectroscopy (EIS) measurements were performed in the frequency range between 20 kHz and 10 Hz, using an imposed AC rms amplitude of 7 mV peak-to-peak. The EIS experimental data was evaluated for its compliance with Kramers–Kronig (KK) criteria by fitting the AC response of the system to the admittance representation of a theoretical circuit containing a ladder of *n* RC elements in series, with an additional capacitance and/or inductance in parallel to the ladder structure, using the software developed by Boukamp.^28^ The choice of the aforementioned equivalent circuit relies on the blocking nature of the electrodes under study (the impedance increases to infinity as frequency approaches zero), which renders the Voigt-type approximation inappropriate.^28^ The compliance with KK criteria was assured for all data by the values of the relative residuals, calculated to be less than 0.5% for both the real and imaginary parts of the impedance and the chi-square parameter which was found to be on the order of 10^–7^ for the complete data series. All of the experiments were conducted inside a faraday cage.

### 2.3 Calculation of Capacitance from EIS Measurements

Capacitance was extracted from the EIS data by adopting the graphical approach developed by Orazem and co-workers for systems exhibiting frequency dispersion effects.^29^ The value of the constant phase exponent, α, was calculated by performing a linear fit to the plot log *Z*_im_ vs log *f*, where *Z*_im_ and *f* represent the imaginary part of the total impedance in Ω and the applied frequency in Hz, respectively. The effective capacitance, *C*_eff_, was then calculated at each frequency using the following equation
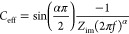
1The final capacitance values, *C*, were determined by averaging the obtained *C*_eff_ values in the frequency range within which variations smaller than 0.2 μF cm^–2^ were recorded (linear portion of the *C*_eff_ vs *f* plot).

### 2.4 Contact Angle Measurements

Contact angle values were extracted from the recorded images based on the gradient of the droplet edge in close proximity to the baseline. Image processing was performed in MATLAB (MathWorks Inc., Natick, MA). At first, the background was subtracted using the built-in Canny edge detection algorithm. Subsequently, the resulted arc (representative of droplet edge) was fitted to a circle equation by means of the incorporated Levenberg–Marquardt nonlinear squares fitting algorithm. The contact angle was then extracted using the calculated coefficients of the fitted equation and by applying the formula
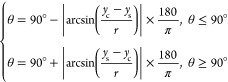
2where *y*_c_, *y*_s_, and *r* are the *y* coordinates of the center of the circle, its projection relative to the contact line, and the radius of the droplet, respectively.

## 3 Results and Discussion

### 3.1 Liquid|Air Electrowetting

#### 3.1.1 Effect of Electrolyte Concentration on the Electrowetting Response of Graphite

**Figure 1:**
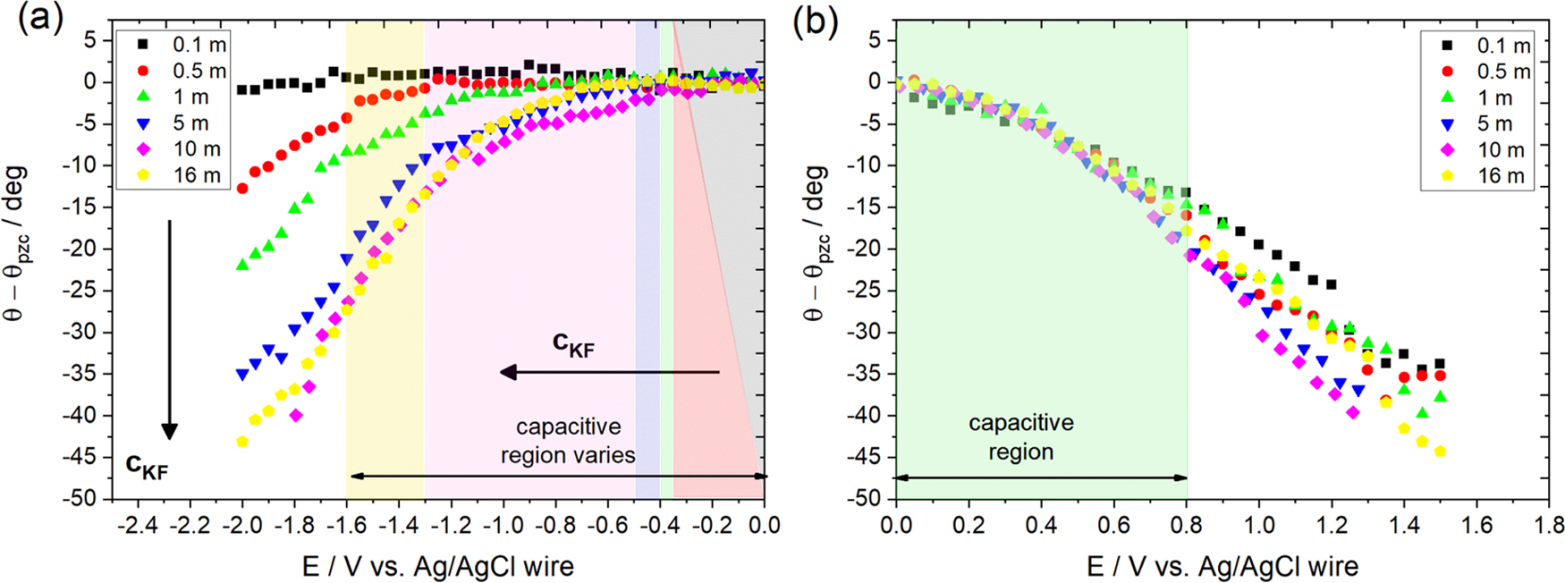
(a, b) Change in the apparent equilibrium electrowetting contact angle, θ, (relative to the value θ_pzc_ at *E*_pzc_) with the applied bias (values are reported vs Ag/AgCl wire) as a function of the electrolyte concentration, *c*_KF_. *E*_pzc_ is located at ca. −0.05 V in the 0.1 to 10 m concentration range and shifts to ca. −0.25 V for the 16 m solution (see Figure S4 and the relevant discussion in the SI). The highlighted regions correspond to the purely capacitive potential window for each *c*_KF_ (changes in color in panel (a) follow those of the data points) as determined via EIS by monitoring the dependence of the phase angle between the AC voltage and current perturbation on the applied DC bias (see Figure S3). Measurements were conducted under static conditions based on the protocol described in the Electrochemical Measurements section.

The CA of the electrolyte droplet deposited on the HOPG was monitored as a function of the potential, *E*, applied vs the Ag/AgCl pseudo-reference electrode using the setup displayed in Figure S1a and applying the experimental protocol described in the Methods section. Figure 1 shows electrowetting curves of the equilibrium CA within a potential window of 3.5 V for KF concentrations, 0.1 m ≤ *c*_KF_ ≤ 16 m. The first notable feature is the change of the apparent CA up to a maximum value of ca. 45° vs its equilibrium value at *E*_pzc_, denoted θ_pzc_ (located at ca. −0.05 V for 0.1 m ≤ *c*_KF_ ≤ 10 m and at ca. −0.25 V for *c*_KF_ = 16 m; see Figure S4 and the relevant discussion in the SI). The second feature of merit is the dependence of the electrowetting response on electrolyte concentrations. For *E* < *E*_pzc_, a strong dependence on *c*_KF_ is observed, while for *E* > *E*_pzc_, the CA hardly varies with the concentration except for the largest positive potentials applied. This indicates a strong asymmetry in the electrowetting curves relative to *E*_pzc_, which increases with decreasing electrolyte concentrations. Similar asymmetry between the electrowetting response for positive and negative biases has been reported in the literature for EWOD^19,30,31^ systems and is mostly attributed to the breakdown of the dielectric layer, which leads to charge trapping in, or on, the insulating film.^21^ Asymmetric electrowetting has been also detected when semiconducting substrates are used with or without a dielectric layer due to space–charge effects in the semiconductor.^10^ In our configuration, however, the absence of a dielectric layer and the semimetallic character of graphite suggest that distinct mechanisms are at play.

**Figure 2:**
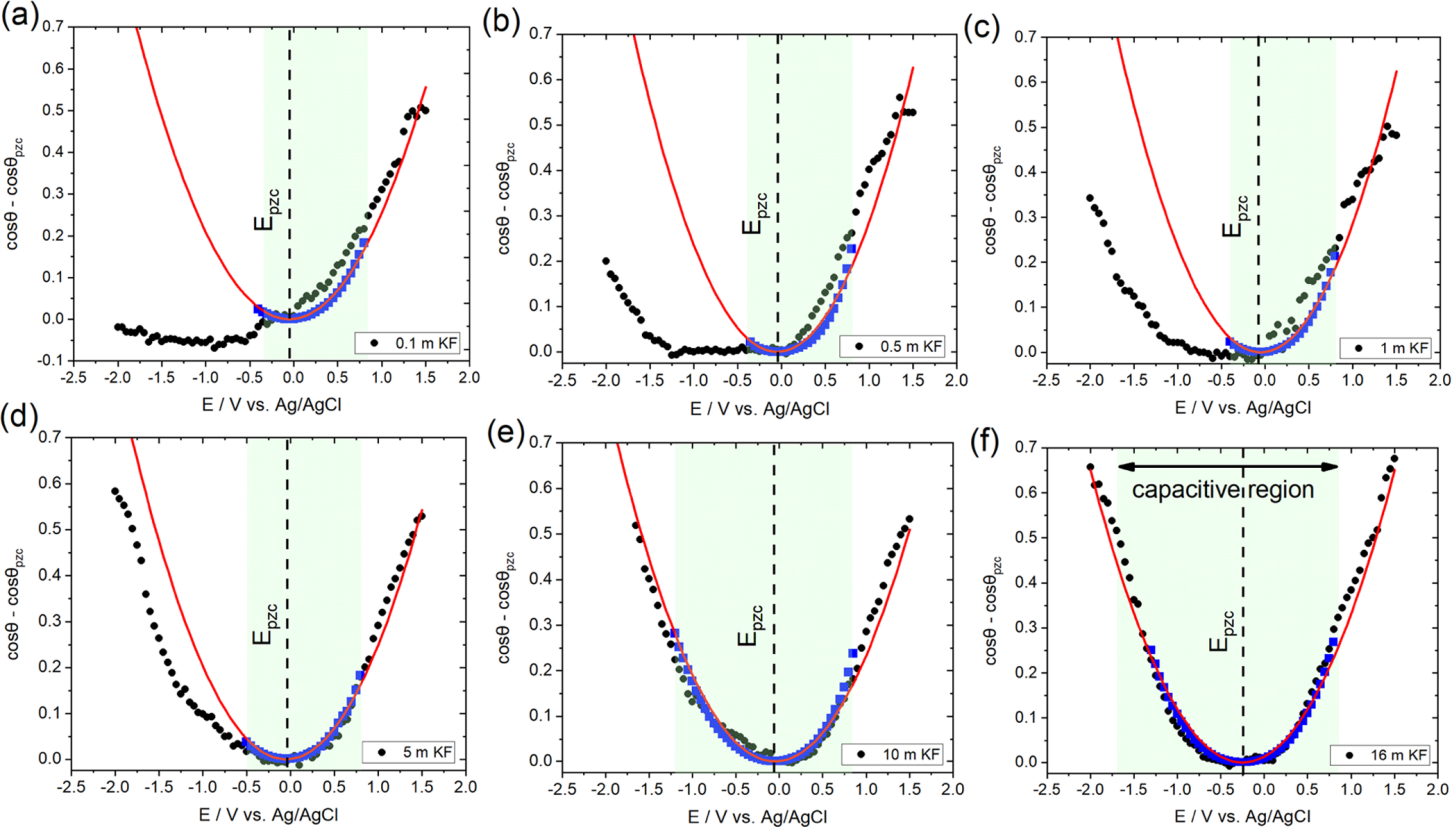
Electrowetting curves based on the experimental data presented in Figure 1a (black dots) for (a) 0.1 m, (b) 0.5 m, (c) 1 m, (d) 5 m, (e) 10 m, and (f) 16 m KF solutions. The values of interfacial tension and capacitance were independently measured for each *c*_KF_ using the pendant drop method (see the Methods section and data in Table S1) and EIS measurements (see Figure S4 and Section S2.2 in the SI), respectively. From these values, cos θ – cos θ_pzc_ was calculated in the purely capacitive window for each *c*_KF_ using the Young–Lippmann equation (blue squares). The theoretical response in the whole potential window studied (red lines) was approximated by fitting a quadratic function of the potential bias to the experimentally measured parameters (cos θ – cos θ_pzc_, see main text and Section S2.3 in the SI). The highlighted region corresponds to the purely capacitive potential window as determined via EIS by monitoring the dependence of the phase angle between the AC voltage and current on the applied DC bias (see Figure S3).

We start by quantifying the departure of our measured electrowetting curves from the ideal electrowetting response captured by the Young–Lippmann (Y–L) equation,^6^
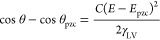
3where *C* and γ_LV_ are the capacitance of the interface and the surface tension of the liquid|vapor interface, respectively. Figure 2 shows a direct comparison between theoretical electrowetting curves estimated using the Y–L equation (eq 3) for each value of *c*_KF_ and the corresponding experimental data presented in Figure 1. The electrowetting response (blue squares in Figure 2) was estimated from the experimentally measured values of γ_LV_ and *C* for each *c*_KF_. The variation of *C* with *E* for each concentration was obtained within the capacitive potential window indicated by green shading in Figure 2. The prediction of the Y–L curve in the whole potential window studied (red lines in Figure 2) was obtained by fitting a quadratic function of *E* to the electrowetting response (blue squares in Figure 2), thus yielding an effective capacitance independent of *E* (see Section S2.3 in the SI). For *c*_KF_ = 10 and 16 m, the experimental response is closely captured by the Y–L equation over the entire potential window, while for *c*_KF_ ≤ 5 m, the experimental data diverges from the theoretical curves for negative potential biases. In fact, the electrowetting effect is considerably reduced as the electrolyte concentration is decreased, and for *c*_KF_ = 0.1 m, the droplet does not exhibit measurable spreading. In contrast, for positive potential biases, the experimental data remains consistent with the Y–L equation for all values of *c*_KF_ investigated. Considering that γ_LV_ is constant for a given value of *c*_KF_ and that the potential window for the static measurements is the same for all electrolyte concentrations, Figure 2 indicates that physicochemical processes occur on the surface of the HOPG that affect the surface charge, at least upon negative polarization.

**Figure 3:**
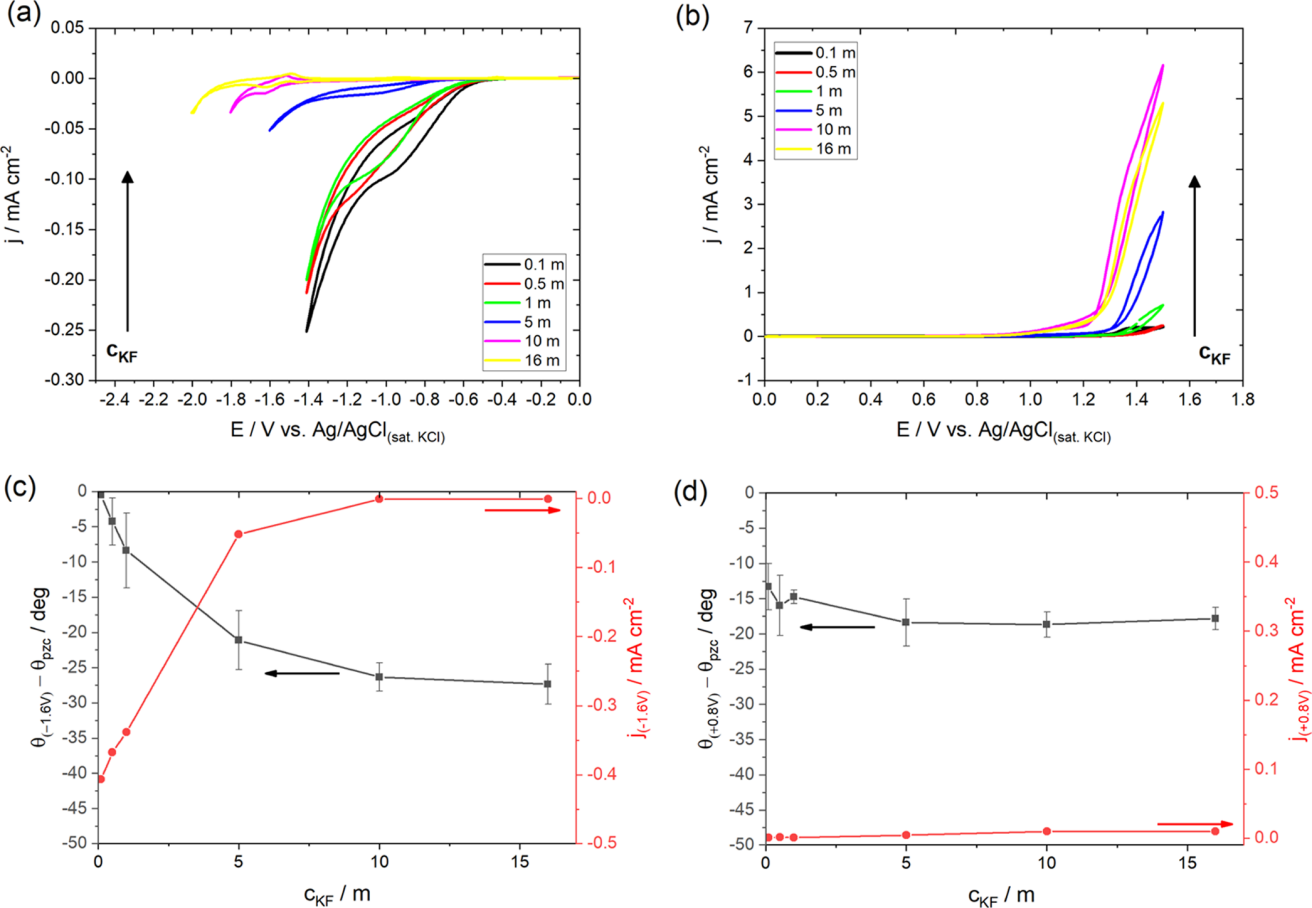
(a, b) Cyclic voltammograms recorded on freshly cleaved HOPG electrodes using the Teflon cell setup (see Figure S1b and Section S1.3 in the SI) in the potential range between (a) 0 up to −2 V vs Ag/AgCl_(sat. KCl)_ and (b) 0 to +1.5 V vs Ag/AgCl_(sat. KCl)_ at a scan rate of 1 V s^–1^ in KF solutions within the concentration range used for the electrowetting measurements. (c, d) Apparent equilibrium electrowetting contact angle, θ, relative to θ_pzc_ and current density, *j*, for each *c*_KF_ at applied potentials (c) −1.6 (θ_(−1.6 V)_ and *j*_(−1.6 V)_) and (d) +0.8 V (θ_(+0.8 V)_ and *j*_(+0.8 V)_) vs Ag/AgCl wire and Ag/AgCl_(sat. KCl)_, respectively. The applied potential values correspond to the negative and positive potential limits of the largest purely capacitive window among the whole *c*_KF_ range investigated (corresponding to the 16 m KF concentration). The results demonstrate the negative effect of high current densities on the electrowetting response at −1.6 V (*E* < *E*_pzc_) and the uniform response among all *c*_KF_ at +0.8 V (*E* > *E*_pzc_) where the rate of the charge transfer processes is low.

Because of the possibility of charge transfer (faradic) reactions on the surface of conducting substrates, the current in the applied potential window was monitored by cyclic voltammetry (CV) and the results are presented in Figure 3a,b. Upon negative polarization (Figure 3a), a decrease in *c*_KF_ results in an increase in current density (per electrode nominal area), *j*. A similar finding has been reported in the literature for studies involving highly concentrated electrolyte solutions and is attributed to the suppression of a hydrogen evolution reaction (HER) due to the decrease of the water-to-electrolyte molar ratio.^24,32,33^ By monitoring *j* vs *c*_KF_ at −1.6 V (corresponding to the negative limit of the largest purely capacitive window, which was determined for 16 m KF), a significant decrease is recorded as *c*_KF_ increases up to 16 m (see Figure 3c). This can be attributed to the high rate of HER at −1.6 V for *c*_KF_ < 5 m. Considering the relatively minor differences in the pH of the electrolytes for *c*_KF_ < 5 m (see Table S1), HER is not expected to occur for *E* > −1.36 V for the 1 m KF and for *E* > −1.3 V for 0.1 m KF,^34^ and hence, further investigation into the processes occurring outside the HER potential region is required. In the voltammograms of Figure 3a, a reductive process is observed before HER (half-wave potential of ca. −0.8 V), which decreases as *c*_KF_ increases. We assign this process to an oxygen reduction reaction (ORR) because graphite is known to exhibit strong affinity to oxygen species present in the atmosphere and/or dissolved in the solution that reduce to H_2_O and/or H_2_O_2_ upon negative polarization.^34−36^ The large surface-to-volume ratio of the droplet due to its small size (less than 200 μm; see Section S2.7 in the SI) is expected to increase the dissolution rate of oxygen from the atmosphere in the electrolyte (edge effects in oxygen diffusion will be also enhanced). Furthermore, the small electrode area under the droplet will practically behave as a microelectrode/sensor, and hence, the sensitivity toward dissolved oxygen will be relatively high (high signal-to-noise ratio). The suppression of the process with increasing electrolyte concentration can be explained by the salting-out effect on oxygen solubility^37^ and the decrease in the diffusion coefficient of oxygen (through the Stokes–Einstein equation)^37^ as a consequence of the significant increase in the solution viscosity (up to 2 orders of magnitude for the 16 m case^32^). Furthermore, electrochemically induced surface processes such as the reduction of quinone groups to hydroquinone (similar to natural graphite and glassy carbon^34,36^) occurring within the ORR potential window may also contribute to the total current density. Such reactions are expected to involve the solvent as a reactant, and hence, a decrease in the water-to-KF molar ratio (as *c*_KF_ increases) is anticipated to decrease the rate of the process in a way similar to the suppression of HER.

The variation of *c*_KF_ leads to opposite trends in *j* (Figure 3a) and the CA (Figure 1a). This implies that faradic reactions at the HOPG surface suppress the electrowetting response, a phenomenon that is enhanced when the rate of charge transfer reactions is increased. We attribute this finding to the compensation of charge on the electrochemical interface arising from the charge accumulation on the HOPG as a result of the several charged electroactive species being involved in ORR^38,39^ and other surface reactions.^34,36^ Additionally, in the case of low electrolyte concentrations (*c*_KF_ ≤ 1 m), as *E* is decreased in the potential region where HER dominates, nanobubbles of hydrogen are formed on the surface of the HOPG^40^ that further increase the potential drop in the vicinity of the substrate electrode.^20^

**Figure 4:**
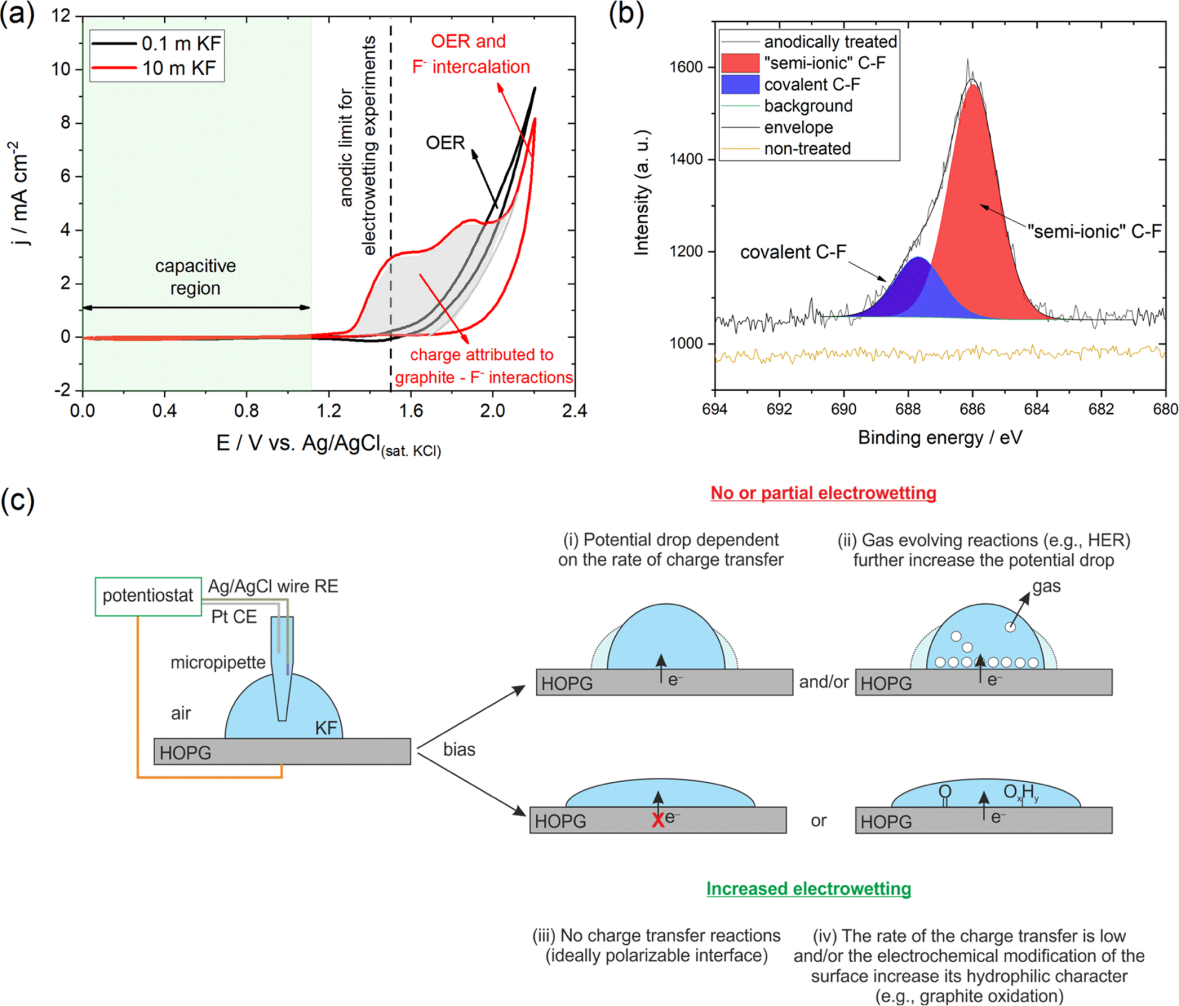
(a) Cyclic voltammograms recorded on freshly cleaved HOPG electrodes using the Teflon cell setup (see Figure S1b and Section S1.3 in the SI) in the potential range of 0 to +2.2 V vs Ag/AgCl_(sat. KCl)_ at a scan rate of 1 V s^–1^ in 0.1 and 10 M KF solutions. (b) XPS narrow window scans of the F1s core levels for the anodically treated and nontreated HOPG samples in 10 m KF following the experimental protocol described in Section S2.4 in the SI. (c) Schematic representation of the processes underlying the electrowetting response of graphite.

Figure 3b shows the CVs recorded within the positive potential range (*E* > *E*_pzc_) of the static measurements of Figure 1b. In contrast to the case of *E* < *E*_pzc_, *j* increases with *c*_KF_ indicating that a reaction involving electrolyte ions occurs upon positive polarization. Malchik et al.^33^ report a similar trend for mixed Chevrel Phase Mo_6_S_8_/Ti_3_C_2_ electrodes in aqueous LiCl at concentrations between 2 and 14 m, where the apparent increase in *j* with *c*_LiCl_ is attributed to the electrooxidation of Cl^–^. To clarify the origin of the recorded anodic currents, we extended the potential window within the OER range, i.e., up to +2.2 V. In the results presented in Figure 4a, an anodic oxidation wave between ca. +1.2 and +2 V is recorded for the 10 m KF solution in contrast to the significantly reduced currents for 0.1 m KF within the same potential region. An investigation of the current density dependence on the scan rate, *v*, for the 10 m KF solution (Figure S6) gives rise to a linear relation between *j* (determined at +1.8 V) and *v*^1/2^. This implies a diffusion-controlled reaction that supports the assumption of a process where F^–^ anions are actively involved. To further investigate the nature of the oxidative process seen at *E* > + 1.2 V for the highly concentrated KF solutions, we performed XPS measurements on anodically treated HOPG electrodes in 10 m KF (Figure 4b), following the protocol described in Section S2.4 in the SI. The spectral trace was fitted with two peaks at 686.0 and 687.7 eV, which are assigned to “semi-ionic” and covalent C–F bonds, respectively, on the basis of previous reports.^41−43^ The bonds described as semi-ionic are attributed to the interaction of fluorine orbitals with the delocalized π-electron system in graphite.^44^ To exclude the presence of residual KF on the surface of the HOPG, high-resolution spectra of the K2p region were also recorded (Figure S7). The absence of a substantial peak assignable to potassium confirms the formation of a chemically bonded fluorine functionality, which is exclusive of potassium. Based on these findings, we conclude that the observed anodic process is due to the formation of fluorine–graphite intercalation compounds (GIC) at a relatively low degree of surface coverage (see Section S2.4 in the SI).

Returning to the results of the electrowetting curves recorded in the positive potential range (Figure 1b), it is seen that the electrowetting response coincides for all *c*_KF_ within the potential region where no faradic reactions occur. Figure 3d shows the dependence of *j* and apparent CA at +0.8 V (corresponding to the positive limit of the purely capacitive window for all *c*_KF_) on *c*_KF_. It is seen that the CA exhibits insignificant variations with *c*_KF_. When exceeding the purely capacitive window, variations appear among different *c*_KF_, which however are not as significant as those seen at negative potentials (Figure S8). We found that graphite becomes more hydrophilic with the increase in *E* for all *c*_KF_. Based on the above analysis of the processes occurring on the surface of the HOPG upon positive polarization, we can conclude the following. (i) At the low concentration regime (*c*_KF_ ≤ 1 m), the application of potentials higher than ca. +0.8 V leads to the electrochemical oxidation of graphite and thus the introduction of oxygen-containing groups. The latter are known to increase the hydrophilic character of graphite,^45,46^ which can explain the observed electrowetting response. (ii) For *c*_KF_ ≥ 5 m, it seems that the formation of the fluorine GIC surface layer alters the surface of the HOPG; however, this modification does not hinder the evolution of CA. Furthermore, as seen from the XPS survey scan in Figure S7a, HOPG is partially oxidized within this potential range, which is expected to promote electrowetting in a similar way to the lower *c*_KF_. The effect of the interfacial physicochemical processes on the electrowetting response of graphite is schematically illustrated in Figure 4c.

#### 3.1.2 Effect of Electrolyte Identity on the Electrowetting Response of Graphite

Having characterized the physicochemical processes at play with KF, we turn to investigating specific ion effects on the electrowetting curves. We focused on *E* < *E*_pzc_ and the highest *c*_KF_ because (i) enhanced interactions between the cations and the electrode are expected,^47^ (ii) the purely capacitive window is expanded allowing for more reliable measurements, and (iii) in this concentration range, no asymmetry about *E*_pzc_ was observed in the electrowetting curves. In this respect, we used CsF as an electrolyte due to its high solubility limit and the larger size of Cs^+^ compared to K^+^.

**Figure 5:**
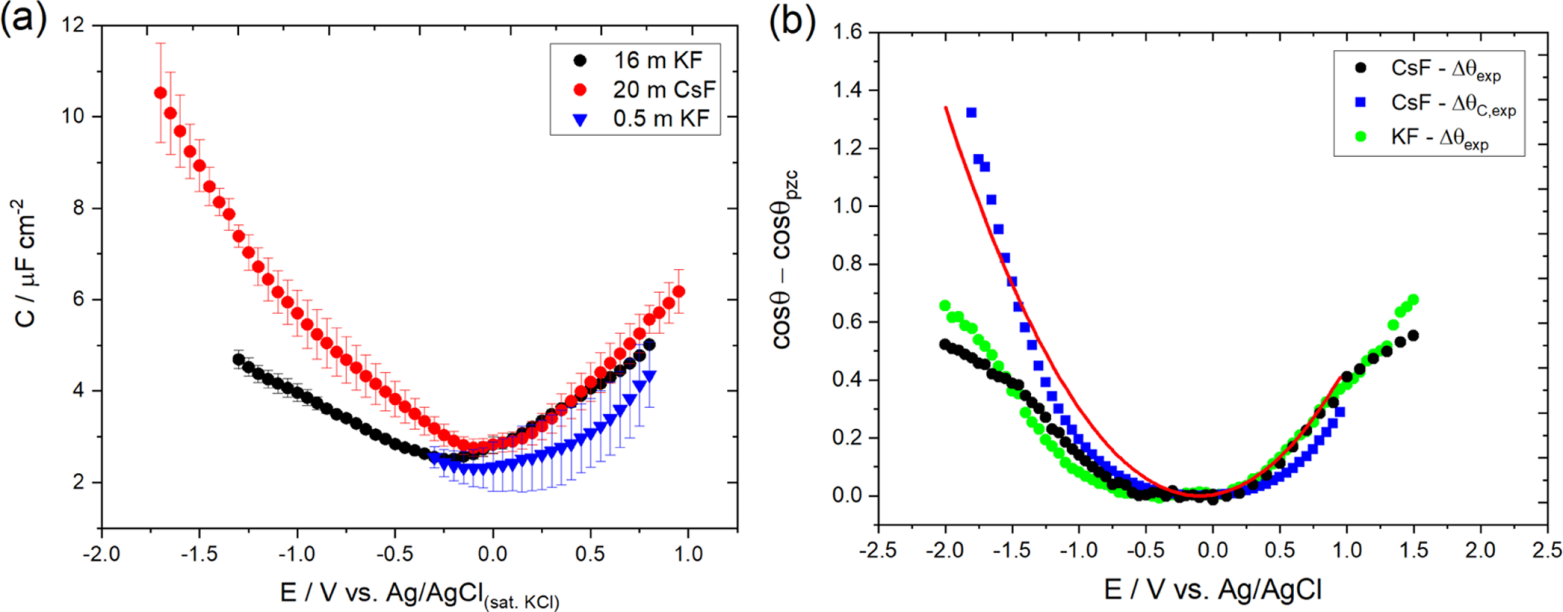
(a) Dependence of HOPG capacitance on the applied potential for 16 m KF and 20 m CsF solutions. For reference, the capacitance of the 0.5 m KF is also given, to highlight the relatively small differences in capacitance with electrolyte concentrations. Capacitance values were extracted from EIS measurements (see Figures S3 and S10) adopting the approach described in the Methods section. EIS spectra were recorded in the frequency range between 20 kHz and 10 Hz with an imposed AC rms amplitude of 7 mV peak-to-peak. The depicted data shows averages and standard deviations of no less than three experiments and corresponds to the potential window within which no faradic reactions occur. A capacitive response was considered for phase angle values higher than 85° (see Figures S3 and S10). The experiments were performed using the Teflon cell setup (see Figure S1b and Section S1.3 in the SI) on freshly cleaved samples. (b) Electrowetting curves based on the experimental data presented in Figure S9e, Δθ_exp_, for a 20 m CsF solution (black dots). For comparison purposes the same data for the 16 m KF solution (Figure 2f) is also given (green dots). The capacitance from panel (a) and the measured interfacial tension (see Table S1) were used to predict the values of cos θ – cos θ_pzc_ (Δθ_C,exp_) through the Young–Lippmann equation (blue squares). The theoretical response in the whole potential window studied (red line) was approximated by fitting a quadratic function of the potential bias to Δθ_C,exp_ (see Section S2.3 in the SI).

Figure 5a shows the capacitance of the interface measured for 16 and 20 m KF and CsF, respectively. The increase in capacitance at *E* < *E*_pzc_ for CsF compared to KF is in line with theoretical calculations for graphene^47^ and experimental measurements for dilute solutions of alkali metal electrolytes on the basal plane of graphite.^48^ In the same figure, the capacitance plot of the 0.5 m KF is also given as a reference. Interestingly, it appears that the capacitance varies only by a factor of less than 1.2 when the electrolyte concentration increases from 0.5 to 16 m KF, implying a relatively weak effect of the ions’ concentration on the total capacitance of the interface. A similar effect has been also reported in the literature for graphite electrodes in aqueous NaF solutions within the concentration range from 10^–5^ to 0.9 M.^49^ The prediction of the electrowetting response (blue squares in Figure 5b), which uses eq 3 with the measured capacitance values as a function of *E* and γ_LV_ (see Table S1) for CsF, suggests that the observed change in capacitance at *E* < *E*_pzc_ is sufficient to distinguish the Y–L curve for CsF from that of the KF solution. Also, the quadratic fit (red line) is less accurate than that for KF in Figure 2, indicating a measurable effect of the variation of the capacitance with a potential. However, this is not measurably reflected in the experimental electrowetting curves (black points in Figure 5b). Zhan et al.^47^ investigated the specific ion effects on the graphene/aqueous alkali metal electrolytes interface by first-principles/continuum simulations, where they showed that large polarized cations exhibit a significant degree of charge transfer from graphene upon their adsorption. On this basis, part of the excess charge used to fill the electronic states of graphene is transferred to the cation, leading to a decrease in the overall potential response of the interface. In particular, the overall charge transfer obtained for Cs^+^ is found to be 27.5 times higher compared to K^+^. Assuming that a similar cation-dependent charge transfer occurs with graphite, we attribute the observation that the capacitance changes are not reflected in the electrowetting behavior to the larger potential drop at the interface in the presence of Cs^+^ ion adsorption compared to K^+^. Changes in the potential of the interface are expected to have a larger influence on the electrowetting response compared to capacitance since the Y–L equation dictates that the difference between the cosines of θ and θ_pzc_ shows a quadratic dependence on *E*. The assumption of such specific cation adsorption processes is supported by the CVs presented in Figure S5. The observed features are in line with those reported recently by Yasuda et al. on graphene^50^ and demonstrate the specific adsorption/desorption of K^+^ and Cs^+^ on HOPG in the high-concentration regime (see also the relevant discussion in Section 2.4 of the SI). Furthermore, the chaotropic effect induced by these cations (due to their strong adsorption on the surface of the electrode)^51^ as well as the bulk properties of these electrolytes in the high-concentration regime (i.e., the strong hydrogen bonds between the F^–^ ions and water)^52^ are considered to be the main factor hindering the HER, hence the resultant expansion of the potential window in the negative side (see Figure 3a). This is in contrast to what is reported in the literature for other highly concentrated aqueous electrolytes, such as LiTFSI, where the expansion of the potential window in the cathodic side is attributed to the water-catalyzed decomposition of the TFSI^–^ anion and the formation of a passivating layer on the surface of the electrode.^53^ In our case though, a similar process is excluded due to the nature of the inorganic salts used and the activity retention of the electrode as seen in the stability of the CVs presented in Figure S5a during cycling (Figure S5b,c).

#### 3.1.3 Dynamic Measurements

**Figure 6:**
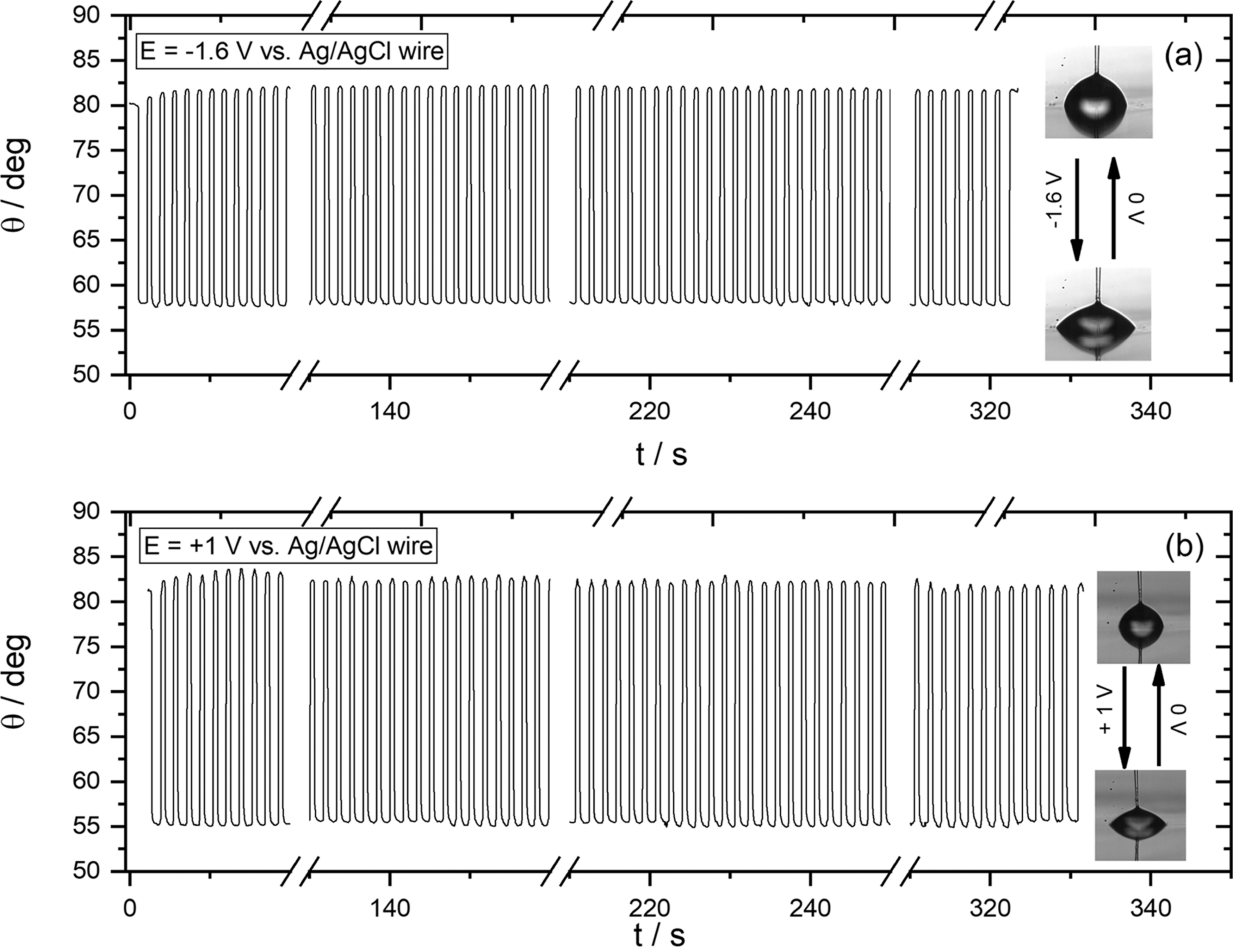
Changes in the apparent contact angle, θ, during wetting/dewetting cycles following the protocol described in the Methods section for a 10 m KF droplet on the HOPG in air. One cycle corresponds to two consecutive potential pulses from (a) 0 to −1.6 V and (b) 0 to +1 V vs Ag/AgCl wire. The potential values lie close to the limits of the purely capacitive window as that determined for the 10 m KF solution (i.e., the rate of charge transfer processes is negligible; see Figures 3, 4, S3, and S6). The changes in the contact angle were monitored using a frame rate of 50 fps, and the duration of the potential pulse was 500 ms. Thus, the time scale corresponds to 200 consecutive wetting/dewetting cycles (see videos in the SI). The data demonstrate the high reproducibility of the phenomenon among several cycles.

Following the investigation of electrowetting on graphite under static conditions, we expand our study by probing the dynamics of the electrowetting response at the air|aqueous electrolytes|graphite interface. At first, we systematically examine the reversibility of the electrowetting process by monitoring the changes in CA among several consecutive wetting/dewetting cycles using solutions of 10 m KF. The latter system has been proved to exhibit a wide potential window that results in a symmetric relative to *E*_pzc_ electrowetting response following the predictions of the Y–L equation (see Figures 1 and 2). Figure 6 shows the changes in CA recorded during 200 consecutive wetting/dewetting cycles. The potential values chosen lie close to the limits of the purely capacitive window (i.e., the rate of charge transfer processes is not significant) as that determined for the 10 m KF solutions (see Figures 3, 4, S3 and S6). The results demonstrate the significantly high degree of reproducibility of the electrowetting process among several consecutive wetting/dewetting cycles (see videos in the SI).

**Figure 7:**
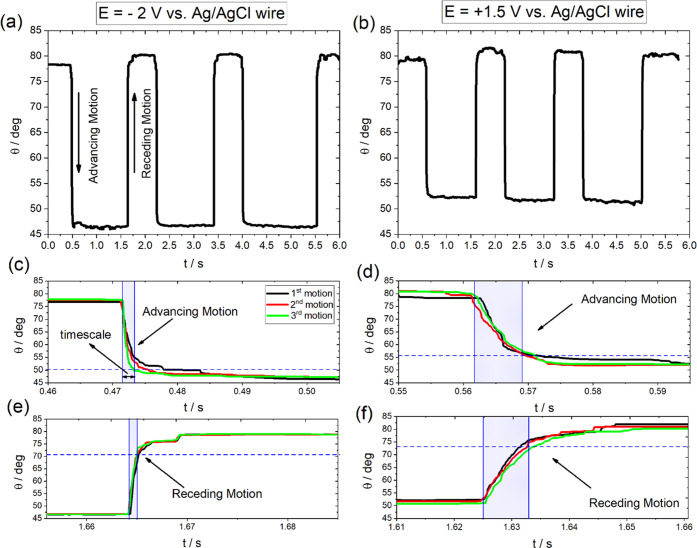
(a, b) Change in the apparent contact angle, θ, during wetting/dewetting cycles following the protocol described in the Methods section in 10 m KF solutions. One cycle corresponds to two consecutive potential pulses from (a) 0 to −2 V (maximum change in θ for *E* < *E*_pzc_) vs Ag/AgCl wire and (b) 0 to +1.5 V (maximum change in θ for *E* > *E*_pzc_) vs Ag/AgCl wire. (c–f) Average timescales (highlighted regions) of advancing and receding motions among consecutive cycles at (c–e) −2 V and (d–f) + 1.5 V vs Ag/AgCl wire. The extracted values for the advancing and receding motions at −2 V are found to be ca. 2.1 (±0.35) and 0.96 (±0.12) ms, respectively. At +1.5 V, the determined values for the advancing and receding motions are 7.5 (±0.2) and 6.63 (±0.12) ms, respectively. The observed increase in θ_pzc_ for the 10 M KF (to ca. 78° from ca. 65° for low *c*_KF_) agrees with what has been recently reported in the literature for aqueous KF solutions up to the solubility limit of the salt.^32^

Subsequently, we aim to provide insights into the effect of the underlying electrochemical processes (when present) on the timescales of the electrowetting response. Figure 7a,b shows the changes in CA recorded during three consecutive wetting/dewetting cycles for 10 m KF with a higher resolution compared to the data presented in Figure 6 (i.e., 6000 and 50 fps, respectively). An interesting characteristic of these curves is the consistency of the recorded contact angle values among the advancing and receding motions of the droplet, which indicates that the occurrence of the underlying processes identified in the previous sections do not degrade reversibility of the electrowetting process. A closer look on the average timescales of the advancing and receding motions of the drop at the specified *E* for each cycle is given in Figure 7c–f. The first noteworthy outcome is that the ratio of the average timescales (among the consecutive cycles) for the advancing and receding motions of the drop at −2 and +1.5 V is ca. 3.4 and 6.9, respectively. This finding suggests that the surface process occurring at the most positive *E* increases the timescales of spreading and receding. This can be explained by the formation of the ionic C–F surface film on graphite for *c*_KF_ ≥ 5 m as evidenced by electrochemical and spectroscopic measurements (see Figure 4a,b). In contrast, at −2 V, no phase formation is observed, and thus, the timescales are faster. Furthermore, by comparing the timescales at a constant potential, it is found that the ratio of the advancing to receding motions at −2 and +1.5 V is ca. 2.2 and 1.1, respectively. These values are ca. 2.3 and 5 times smaller than those previously reported for lower electrolyte concentrations at the applied bias +1.3 V vs *E*_pzc_.^54^ The observed difference is ascribed to the suppression of electrolysis when using highly concentrated electrolytes that decreases the potential drop and hence accelerates the charging process. The latter results in faster timescales for the advancing motion compared to the case of lower electrolyte concentrations. Finally, the relatively faster receding motion at −2 V occurs because of the absence of additional processes (such as phase formation or HER) to the discharging of the double layer upon stepping the potential from −2 to 0 V (see Figure 3).

### 3.2 Liquid|Liquid Electrowetting

Electrowetting at the liquid|liquid interface has found notable applications in commercial optofluidic devices, such as liquid lenses.^14,55^ The latter are optical devices containing two immiscible liquids which serve as an optical medium. In most cases, the electrolytically conducting phase is an aqueous electrolyte and the insulating phase is an organic solvent. Upon application of an external bias, the curvature of the interface changes and the extent of that change along with the difference between the refractive indices of the individual phases determine the optical power (the reciprocal of the focal length) of the lens. Commercially available liquid lenses use a dielectric layer to insulate the conducting substrate (e.g., Au) from the electrolyte solution. In this way, degradation of the substrate and/or the electrolyte due to the occurrence of electrochemically induced reactions, such as surface oxidation, adsorption of impurities on the electrode, and electrolysis, is prevented.^17,19^ Nevertheless, the presence of the insulating layer (with a thickness of hundreds of nanometers often extending to the micrometer range) necessitates the application of high voltages (in the order of tens to hundreds of volts^56^) that consequently increases the energy input of the device. To overcome this issue, alternative approaches have been suggested including the removal of the dielectric layer and the use of immiscible electrolyte interfaces^57^ or relatively inactive substrates.^54^ However, these approaches exhibit wetting hysteresis, electrolyte and/or substrate degradation, a narrow operational window (less than 1 V), and complex organic electrolytes, while in most cases, a potential threshold must be overcome to induce electrowetting similar to electrowetting on dielectrics.

**Figure 8:**
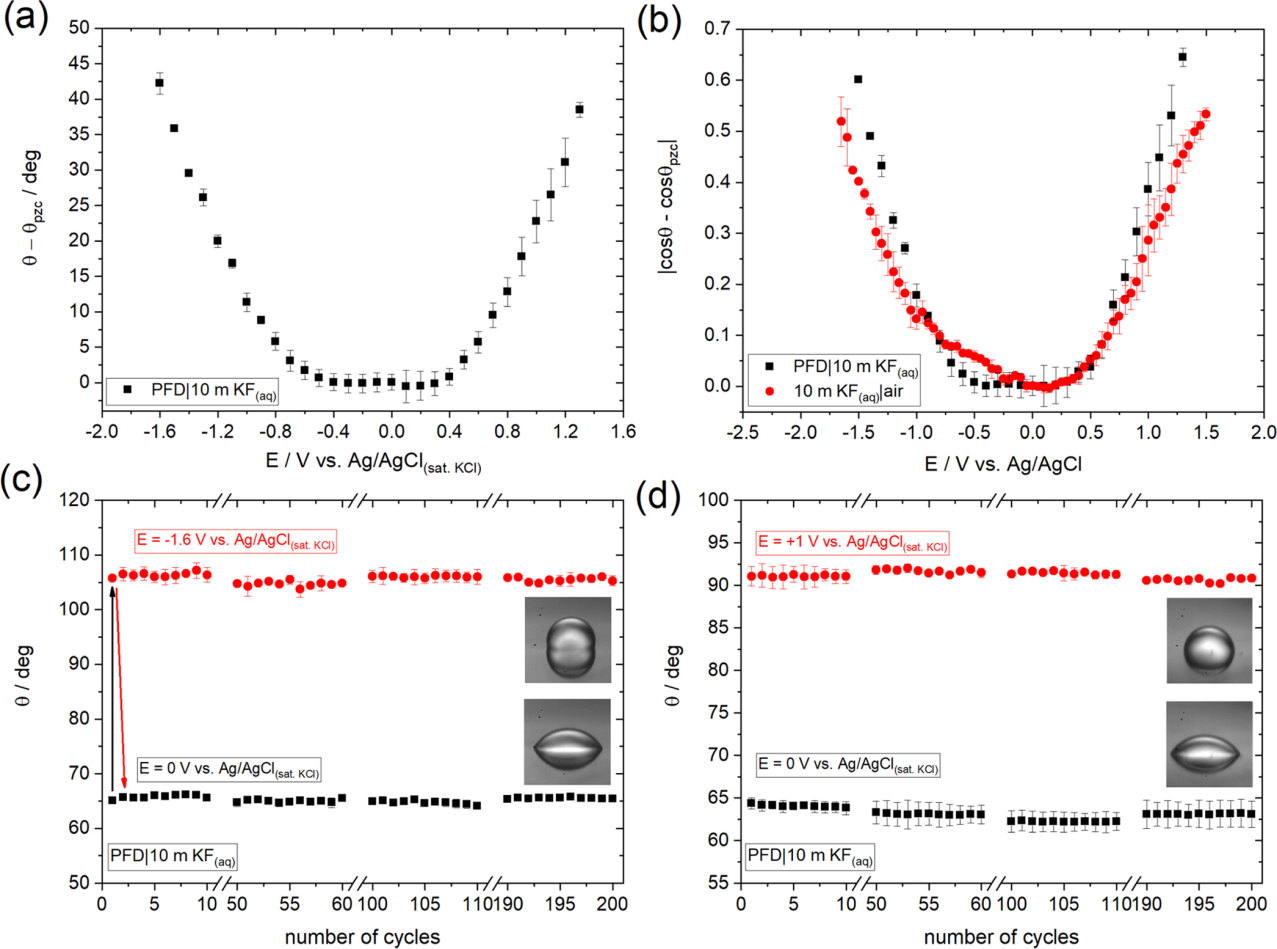
(a) Change (absolute value) in the apparent equilibrium electrowetting contact angle, θ, (relative to the value θ_pzc_ at *E*_pzc_) with the applied bias for the PFD|10 m KF_(aq)_ liquid|liquid interface. Measurements were conducted under static conditions based on the protocol described in the Electrochemical Measurements section. Potential values are reported vs Ag/AgCl_(sat. KCl)_. (b) Electrowetting curve for the PFD|10 m KF_(aq)_ (black squares) based on the experimental data presented in panel (a). For comparison, the same data for 10 m KF_(aq)_ in air is also included (red dots) (see Figure 2e and discussion in Section S1.1). (c, d) Changes in the apparent contact angle, θ, during wetting/dewetting cycles following the protocol described in the Methods section at the PFD|10 m KF_(aq)_ liquid|liquid interface. One cycle corresponds to two consecutive potential pulses from (c) 0 to −1.6 V and (d) 0 to + 1 V vs Ag/AgCl_(sat. KCl)_. The potential values lie close to the limits of the purely capacitive window as that determined for the 10 m KF solution (i.e., the rate of charge transfer processes is negligible; see Figures 3, 4, S3, and S6). The changes in the equilibrium contact angle were extracted by recording the dynamics of the advancing/receding motions using a frame rate of 50 fps (see videos in the SI). The data demonstrate the high reproducibility of the phenomenon among several cycles.

Herein, we introduce an innovative strategy by extending our approach described in the previous sections to the liquid|liquid configuration. We developed a pipette-free liquid|liquid system comprised of a 10 m KF aqueous solution (denoted as KF_(aq)_) as the light phase (ρ = 1.291 g cm^–3^; see Table S1) and the organic solvent perfluorodecalin (PFD) with no added electrolyte as the heavy phase (ρ = 1.917 g cm^–3^). Figure 8a shows the CA variations with *E* vs the Ag/AgCl_(sat.KCl)_ reference electrode for the PFD droplet deposited on the surface of the HOPG both being immersed in a solution of 10 m KF_(aq)_ (see Figures S1c and S19). As for the air|liquid configuration (see Figures 1 and S14), the electrowetting response is symmetric relative to *E*_pzc_ with changes of the apparent CA up to a maximum value of ca. 42° vs θ_pzc_ within a potential window of 2.9 V. Figure 8a also shows that the CA increases with *E* (θ – θ_pzc_ > 0) in contrast with the air|liquid configuration where it decreases (see Figure 1). However, a direct comparison in Figure 8b of the absolute values cos θ – cos θ_pzc_ for 10 m KF_(aq)_ (black squares) and 10 m KF_(aq)|air_ (red circles; reproduced from Figure 2e) reveals a very similar dependence on *E*. The opposite variation in CA stems from the fact that the electrolyte surrounds an insulating droplet in the liquid|liquid configuration, whereas the electrolyte drop is surrounded by an insulator in the liquid|air configuration. Upon application of a potential bias, the substrate–electrolyte surface energy is reduced, resulting in the spreading of the electrolyte. In the liquid|air configuration, this leads to the spreading of the droplet and thus a reduction in CA, whereas in the liquid|liquid configuration, the insulating droplet retracts resulting in an increase in its CA. Moreover, considering that the interfacial surface tension of the 10 m KF_(aq)_|PFD liquid–liquid system (determined to be 82.69 ± 0.7 mN m^–1^; see Section S1.5 in the SI) is very close to that of the 10 m KF_(aq)_|air system (87.69 ± 0.19 mN m^–1^; see Table S1), the variations of θ with respect to θ_pzc_ for the PFD droplet are quantitatively very close to those of the 10 m KF_(aq)_ droplet in air, as shown in Figure 8a.

Furthermore, it can be seen that electrowetting is induced with a potential threshold of less than 800 mV (similar to liquid|air electrowetting), which is 2 times lower to what has been previously reported for liquid|liquid electrowetting on conductors.^54,57^ We attribute this finding to the almost complete wettability of graphite by PFD (as evidenced by a less than 15° CA in air, see Figure S20) in contrast to its increased hydrophobic character in contact with highly concentrated electrolytes (note the high CA for 10 m KF in air, i.e., ca. 80 ± 2° in Figures 6, 7, and S14). In this way, the thin insulating layer previously considered to be present between the droplet and the substrate when organic solvents and aqueous electrolytes are used as a light and heavy phase, respectively (which leads to the increased potential threshold for electrowetting), is excluded.

From the application perspective, reproducibility of the electrowetting phenomenon is critical since it has a direct effect on the overall performance of the device. To investigate the reproducibility of the developed liquid|liquid system, we monitored the changes in CA during 200 consecutive wetting/dewetting cycles. In line with what is reported in Section 1.3, the potential values chosen lie close to the limits of the purely capacitive window for the electrolytically conducting 10 m KF_(aq)_ phase. The results are shown in Figure 8c,d, and they demonstrate the highly reproducible changes of CA among several consecutive wetting/dewetting cycles (see videos in the SI).

Finally, it also worth highlighting the fact that in contrast to the usage of hazardous organic solvents (such as dichlorobenzene,^57^ dichloroethane,^58^ and nitrobenzene^59^) in conventional approaches and commercialized devices,^55^ the solvent used in this study is nontoxic, biologically and chemically inert, and stable up to 400 °C^60^ and thus can be considered as a safe, environmentally friendly option for optofluidic devices.

## 4 Conclusions

We demonstrate that the electrowetting response of graphite is markedly amplified by the use of highly concentrated aqueous electrolyte solutions. The suppression of the underlying faradic processes results in electrowetting behavior that closely follows the Young–Lippmann equation without the use of an insulating overlayer. A fully reversible and reproducible response is attained within an outstanding potential range of up to 2.8 V. A pioneering strategy is introduced to tune and control electrowetting of graphite in contact with aqueous electrolyte solutions, while at the same time, novel insights are provided into the electrowettability of graphite surfaces in the concentration range up to the electrolyte solubility limit. The latter is directly relevant to maximizing the extent of electrolyte accessibility in the carbon-based electrodes that are widely used in state-of-the-art aqueous energy-storage systems. Our work is expected to stimulate new research toward utilizing electrowetting to boost the performance of devices operating on the basis of physiochemical processes at the conductor/aqueous electrolytes interface.

## Supporting Information Available

Experimental section: materials and chemicals, preparation of the electrodes, PTFE cell configuration, physicochemical characterization, surface tension, conductivity, pH and mass density measurements; Results and Discussion section: electrowetting curves, capacitance studies of the graphite–aqueous KF interface, estimation of the electrowetting response based on the Young–Lippmann equation, investigation of the physicochemical processes occurring at the graphite–aqueous KF interface, effect of electrolyte identity on the electrowetting curves, dynamic measurements, optical images of the droplets at selective potential biases for the systems under study (PDF)
Data presented in Figure 6a (MP4)
Data presented in Figure 6b (MP4)
Data presented in Figure 8c (MP4)
Data presented in Figure 8d (MP4)
