# Supplementary material for: Taming Electrowetting Using Highly Concentrated Aqueous Solutions
Source: J Phys Chem C Nanomater Interfaces. 2022 Nov 30;126(49):21071–83. doi: 10.1021/acs.jpcc.2c06517 (PMC9761672; doi:10.1021/acs.jpcc.2c06517)
Supplement: Supplementary file 1 — jp2c06517_si_001.pdf [file jp2c06517_si_001.pdf]

Supporting Information

Taming Electrowetting Using Highly Concentrated Aqueous Solutions

Athanasios A. Papaderakis[a,b], Kacper Polus[a,c], Pallav Kant[d], Finn Box[d], Bruno Etcheverry[d], Conor Byrne[a,c], Martin Quinn[d], Alex Walton[a,c], Anne Juel[d], Robert A. W. Dryfe[a,b]

[a]Department of Chemistry, University of Manchester, Oxford Road, Manchester, M13 9PL, United Kingdom

[b]Henry Royce Institute, University of Manchester, Oxford Road, Manchester, M13 9PL, United Kingdom

[c]Photon Science Institute, University of Manchester, Oxford Road, Manchester, M13 9PL, United Kingdom

[d]Department of Physics and Astronomy, Manchester Center for Nonlinear Dynamics, University of Manchester, Oxford Road, Manchester, M13 9PL, United Kingdom

Correspondence to: athanasios.papaderakis@manchester.ac.uk (A.A.P.), anne.juel@manchester.ac.uk (A.J.), robert.dryfe@manchester.ac.uk (R.A.W.D)

Table of Contents

1. Experimental Section S3

1.1 Materials and chemicals S3

1.2 Preparation of the electrodes S4

1.3 PTFE cell configuration S3

1.4 Physicochemical characterization S4

1.5 Surface tension, conductivity and mass density measurements S4

2. Results and Discussion Section S5

2.1 Electrowetting curves S5

2.2 Capacitance studies of the graphite – aqueous KF interface S5

2.3 Estimating the electrowetting response based on the Young – Lippman equation S7

2.4 Investigation of the physicochemical processes occurring at the graphite – aqueous KF interface.S8

2.5 The effect of electrolyte identity on the electrowetting curves S12

2.6 Dynamic measurements S14

2.7 Optical images of the droplets at selective potential biases for the systems under study S16

2.8 Captions of movie files S21

3. References S22

1. Experimental Section

1.1 Materials and chemicals

HOPG (ZYA quality, mosaic spread - 0.4±0.1°) was purchased from Scanwel, UK. Anhydrous KF (BioUltra ≥ 99.5 % (F)) from Sigma was used for the preparation of the electrolyte solutions in the 0.1 – 10 m range and $KF \cdot 2H_2O$ (98.5 % + purity) from Alfa Aesar for the 16 m concentration. CsF (99.9% metals basis) from Alfa Aesar was used for the relevant electrolyte solutions in the whole concentration range. LiCl (BioXtra ≥ =99%) was purchased from Sigma. Hydrochloric acid (~ 37 % with specific gravity of 1.18) from Fisher Scientific UK Limited, KCl (BioUltra ≥ 99.5 % (AT)) from Sigma Aldrich and agarose also from Aldrich were used for the preparation of the reference electrodes. Ultra-pure water (18.2 MΩ cm resistivity at 25°C, Milli-Q Direct 8) was used in all studies. All solutions were purged with nitrogen prior to the experiments.

1.2 Preparation of the electrodes

HOPG served as the working electrode (WE). Electrical connection was made by stripping an enameled Cu wire (RS components, UK) for about 1 cm in each end and adhering one side to the edge plane of the HOPG with silver conductive epoxy (RS components, UK). After curing for 24 h, the silver epoxy was covered by an insulating resin and left to dry for 3 h. The reference electrode (RE) used for the electrowetting experiments was a Ag/AgCl wire, fabricated by anodization of a stripped Ag wire (99.99% purity, 0.20 mm diameter, coating thickness ca. 0.035 mm from Advent, UK) to each end (ca. 0.5 cm) in 0.5 M HCl. The wire was coated with a thin layer of poly(tetrafluoroethylene), PTFE, to avoid direct contact with the Pt wire inside the micropipette. The procedure involved the application of three consecutive potential pulses at 0.5, 1.0 and 1.5 V, each for 30 min, using a two-electrode configuration with a Pt mesh as the counter electrode (CE). For the cyclic voltammetry and the capacitance measurements, a custom-made Ag/AgCl (sat. KCl) electrode with an agarose gel frit was used. Its detailed preparation procedure can be found in the supplementary material of [1]. A Pt wire was once again used as a counter electrode. In all cases, prior to each measurement the potential of the reference electrode was recorded with respect to a commercially available Ag/AgCl (sat. KCl) electrode (from Sigma) in a saturated KCl solution to exclude the possibility of potential drifts among different measurements.

1.3 PTFE cell configuration

For the cyclic voltammetry and capacitance experiments the setup used is illustrated in Figure S1b. It consists of a PTFE cylinder (of ca. 0.2 cm^3) with a disk-shaped opening of 3 mm diameter. To ensure that no leakage of the solution occurs (especially at very low electrolyte concentrations where the viscosity is significantly decreased), the bottom part of the cylinder was sealed onto the HOPG substrate with a thin (ca. 1 mm) poly(dimethylsiloxane), PDMS, gel layer (Sylgard™ 527, Dow Corning). To set up the cell a smaller cylinder (3 mm in diameter) was mounted through the disk-shaped opening to seal the Teflon cell and leave an annular region ca. 1 mm deep below the PTFE cylinder where the PDMS mixture was carefully injected. The whole assembly was then transferred to an oven where it was kept at 90°C for 2 h to allow the PTFE gel to cure, followed by cooling at room temperature for at least 3 h and removal of the cylinder. The nominal exposed area was confirmed by performing cyclic voltammetry experiments in N_2 purged solutions of the desired electrolyte concentration + 10 mM of potassium ferricyanide (ACS reagent ≥ 99.0 % from Sigma Aldrich) at scan rates of 1, 0.75, 0.5, 0.25, 0.1 and 0.05 V s^{-1} with subsequent analysis of the data by means of the Randles – Sevčik equation [2].

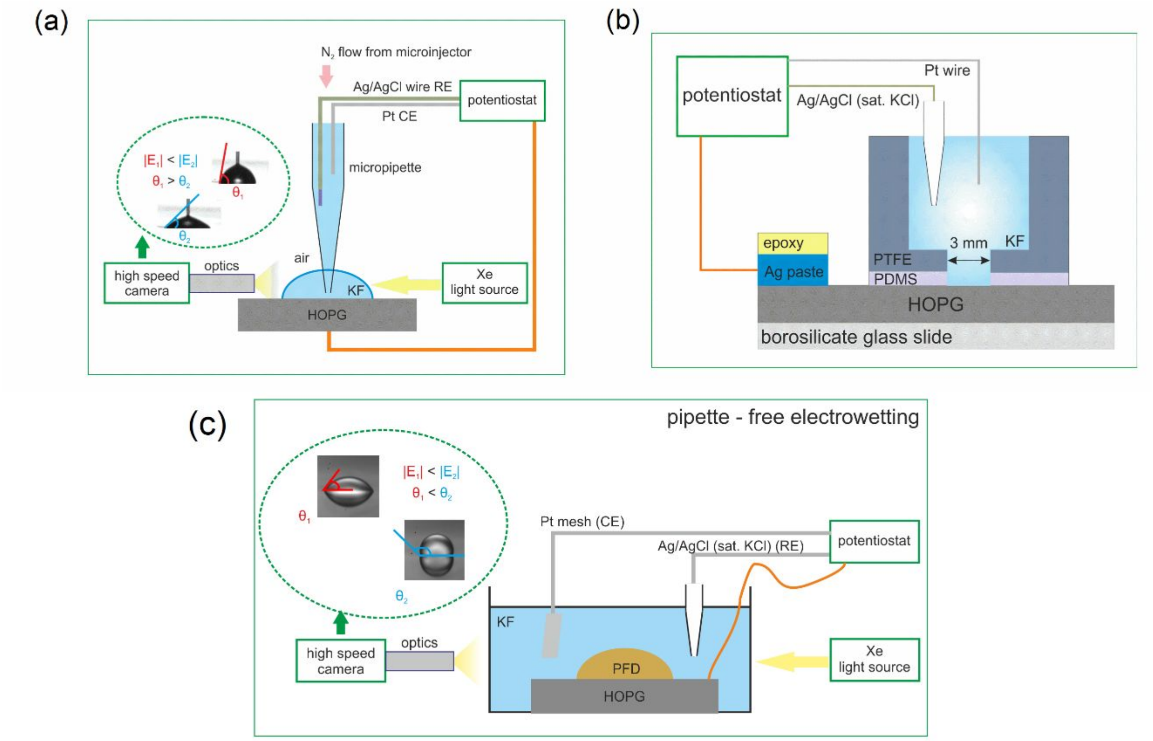

Figure S1. Schematic representation of the setup used for (a) the liquid|air electrowetting experiments, (b) cyclic voltammetry and AC measurements and (c) liquid|liquid electrowetting experiments. For a detailed description refer to the relevant sections in Methods.

1.4 Physicochemical characterization

X-ray photoelectron spectroscopy (XPS) measurements were carried out on a SPECS ultra-high vacuum instrument using a monochromated Al-Kα source (1486.6 eV), micro-focused to a 1 mm analysis spot. The kinetic energy of the photoemitted electrons was analyzed using a SPECS PHOIBOS 150 NAP electron energy analyzer operating at a pass energy of 20 eV. In order to prevent charging artefacts in the acquired spectra an electron flood gun was used during analyses (7 eV, 50 μA). Spectra were processed using CASAXPS software.

1.5 Surface tension, conductivity, pH and mass density measurements

The liquid-air surface tension, γ_{LV}, of the electrolyte solutions in the whole concentration range studied, was determined experimentally by implementing the pendant drop method (droplet volume ca. 15 ± 0.5 μL) using a Theta Optical Tensiometer (Biolin Scientific, Sweden) running OneAttension software version 2.3 and applying the Young – Laplace equation. The specific conductance of the electrolyte solutions, σ, was determined experimentally using an electronic conductivity meter from Mettler Toledo. The pH of the electrolytes was measured using a pH meter from Mettler Toledo (SevenExellence^{TM}). The density of the solutions was calculated by weighting (using a 4-digit high precision analytical scale from Ohaus) a constant volume of each solution, i.e., 1000 μL, collected with an automatic micropipette.

2. Results and Discussion Section

2.1 Electrowetting curves

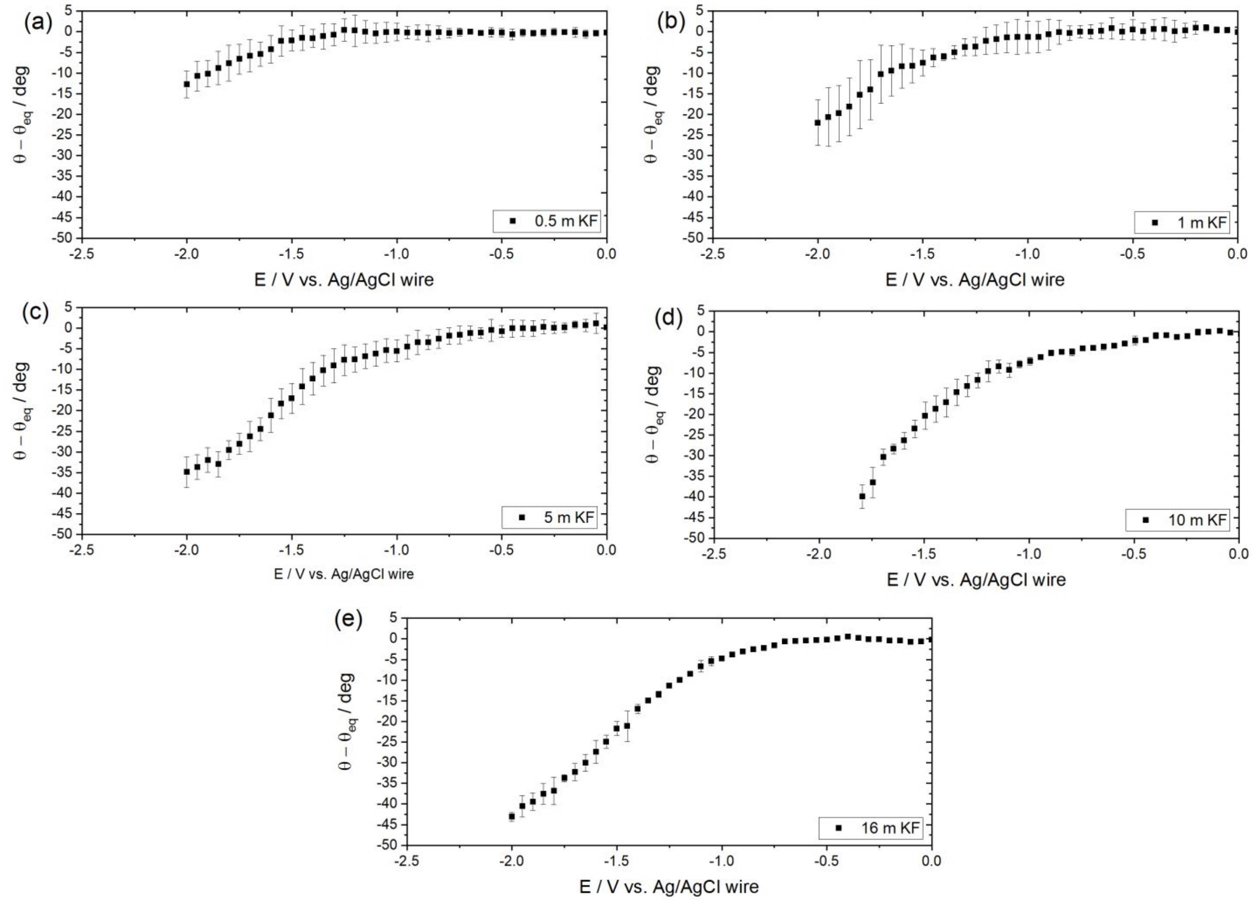

Figure S2. Changes in apparent contact angle, θ, as recorded in the potential range within 0 and -2 V vs. Ag/AgCl wire between (a) 0.5 m, (b) 1 m, (c) 5 m, (d) 10 m, (e) 16 m KF solutions. The results correspond to averages and standard deviations of no less than five experiments on freshly cleaved HOPG. Measurements were conducted under static conditions based on the protocol described in Methods.

Figure S2 shows the dependence of the apparent contact angle, θ, on the applied potential, E, for c_{KF} in the range of 0.5 to 16 m. The standard deviations for all measurements are also included. The data highlights the high degree of reproducibility for c_{KF} > 5 m.

2.2 Capacitance studies of the graphite – aqueous KF interface

The ideally polarizable character of the interface was investigated by means of EIS. The experiments were conducted on freshly cleaved HOPG using the setup of Figure S1b. The recorded impedance spectra are presented in Figure S3 adopting the Bode representation. In general, the AC response of an ideally polarizable (capacitive) interface, i.e., no faradaic reactions occur, exhibits two bend points and changes between a phase angle of 0° at high frequencies (the AC response is attributed to both the ohmic components of the system, e.g., the electrolyte resistance, cell resistance etc. as well as its reactance; the contribution of the latter increases with decreasing frequency) to a constant value of 90° at medium to low

frequencies (the impedance of the capacitor dominates the AC response). The introduction of faradaic processes at the interface leads to charge being transferred through the double layer (leakage capacitor) and deviations from 90° in the Bode phase plots are observed. On this basis, it can be seen from the obtained Bode spectra that as the electrolyte concentration increases from 0.1 to 16 m the purely capacitive potential window increases significantly, from ca. 1.1 to 2.3 V. This is consistent with literature reports for energy storage systems where highly concentrated electrolyte solutions are used to extend the stability window of the system [1], a phenomenon attributed to the suppression of the electrolysis rate with decreasing water content. A noteworthy outcome from the data in Figure S3 is the fact that the deviations from the purely capacitive behavior are mainly detected upon negative polarization. Considering that the asymmetry in the electrowetting curves of Figure 1 is also observed for $E \leq 0$, this finding suggests that the occurrence of faradaic processes on the surface of the substrate are correlated to the retardation of the CA evolution with decreasing c_{KF}.

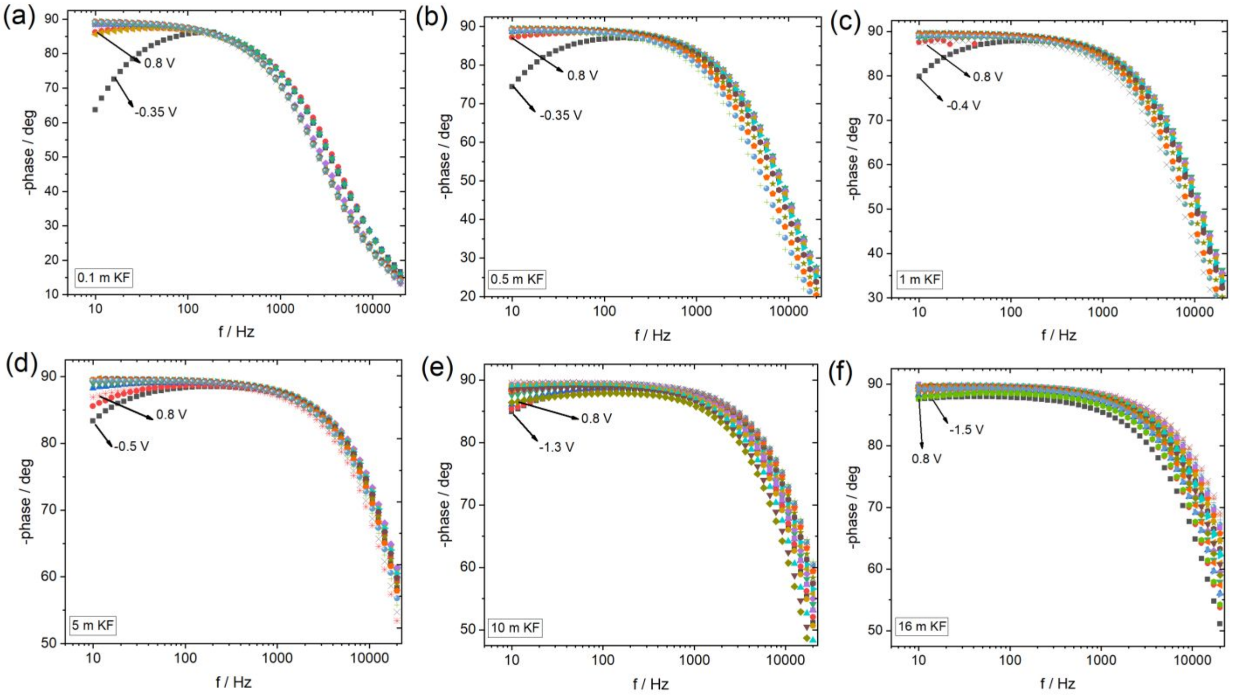

Figure S3. EIS spectra adopting Bode representation in (a) 0.5 m, (b) 1 m, (c) 5 m, (d) 10 m and (e) 16 m KF solutions. Experiments were conducted in the frequency range between 20 kHz and 10 Hz with an imposed AC rms amplitude of 7 mV peak-to-peak. The experiments were performed using the Teflon cell setup (see Figure S1b and section 1.3 herein) on freshly cleaved HOPG samples.

Following the Bode analysis, we extracted the capacitance values by analyzing the AC data of Figure S3 based on the methodology described in Methods. The results are displayed in the C vs. E plots in Figure S4 and correspond to the potential window within which no faradaic reactions occur. It is evident that as the electrolyte concentration increases, the capacitance of the interface displays a symmetrical response with respect to the potential, in line with what has been recently reported by Iamprasertkun et al. for aqueous KF solutions [1]. The minimum of these plots corresponds by definition to the potential of zero charge (E_{pzc}) of the graphite – electrolyte interface [3,4]. Although the minimum is not very sharp for c_{KF} < 5 m (most probably due to the presence of surface states on graphite arising from the adsorption of airborne contaminants in the low electrolyte concentration regime [5]) it can be approximated that E_{pzc} is ca. −50 mV vs. Ag/AgCl$_{(sat.KCl)}$ in the 0.1 to 10 m concentration range. This apparent independence of the E_{pzc} on the electrolyte concentration agrees with previous studies investigating the capacitance of the graphite –

aqueous halides electrolytes interface [3,4]. In the case of the 16 m KF solution a negative shift of −200 mV is observed in the E_{pzc} indicating that fluoride ions are specifically adsorbed on graphite. We attribute this finding to the poorly solvated fluoride ions in the 16 m solution arising from the significantly decreased molar ratio of water to KF (ca. 2.5) [6]. This leads to a shorter distance between the electrolyte ions and the surface of the electrode in the compact layer, which allows for stronger interactions between them.

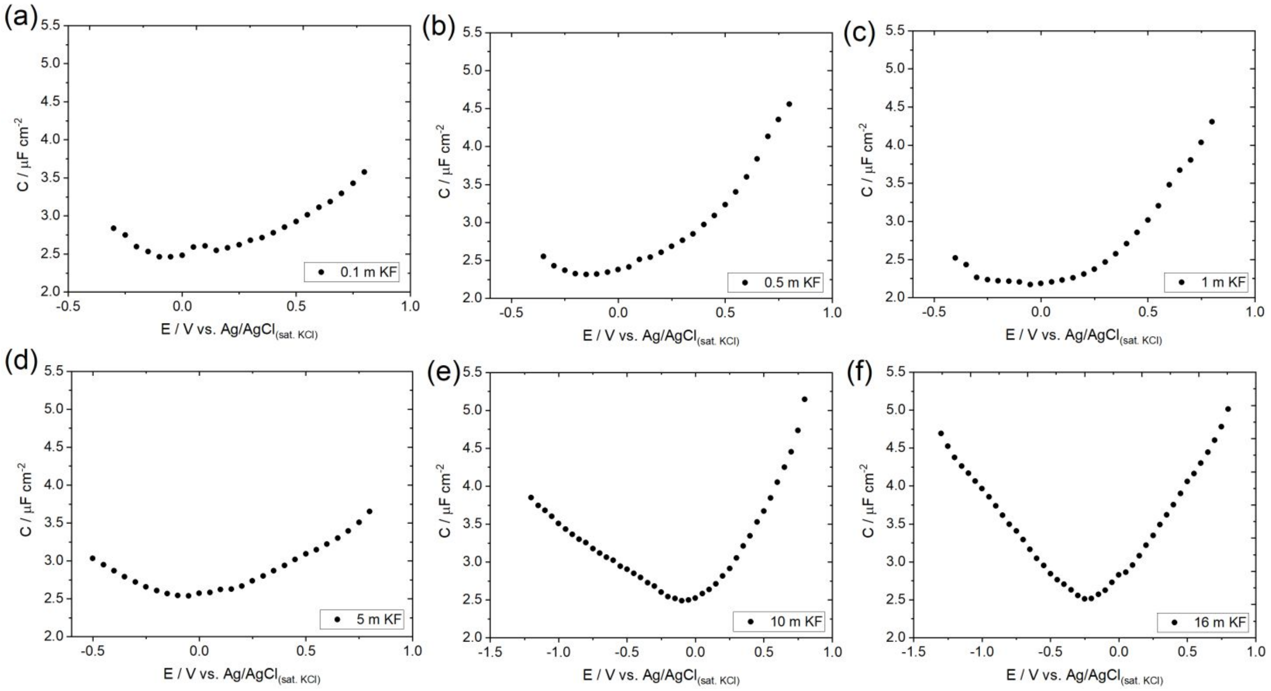

Figure S4. Dependence of HOPG capacitance on the applied potential for (a) 0.5 m, (b) 1 m, (c) 5 m, (d) 10 m and (e) 16 m KF solutions. Capacitance values were extracted from electrochemical impedance spectroscopy (EIS) measurements (see Figure S3) adopting the approach described in the text below. EIS spectra were recorded in the frequency range between 20 kHz and 10 Hz with an imposed AC rms amplitude of 7 mV peak-to-peak. The depicted data shows averages of three experiments and corresponds to the potential window within which no faradaic reactions occur. A capacitive response was considered for phase angle values higher than 85° (see Figure S3). The experiments were performed using the Teflon cell setup (see Figure 1b and section 1.3 herein) on freshly cleaved HOPG samples. Note the extension of the potential axis at negative potentials for the higher electrolyte concentrations.

2.3 Estimating the electrowetting response based on the Young – Lippman equation

The changes in the macroscopic CA with respect to the applied bias are described by the Young-Lippmann (Y-L) equation (also known as the electrowetting equation) [7]; see eq.1 in the main text. In EWOD, C is practically related to the thickness of the dielectric layer and its relative permittivity [7,8] and is considered to be constant (i.e., independent of E) for a specific type of material with a defined thickness. Furthermore, the electrochemical interface is considered ideally polarizable, i.e., no faradaic reactions occur. In the absence of a dielectric layer (EWOC), C is governed by the direct interaction of the electrolyte ions with the electrochemically active substrate, e.g., adsorption processes [9–11], the organization of the ions close to the surface [10,12], the thickness of the double layer governed by the electrolyte nature and its ionic strength [12], diffusion of ions [2] etc. as well as the intrinsic properties of the electrode, e.g. density of states near the Fermi level and the effect of the applied potential on the position of the latter [5,13,14]. All these processes are potential dependent making C a function of the applied bias. In this respect we used the experimentally determined capacitance values of Figure S4 to estimate the changes in CA with the applied potential. Additionally, γ_{LV} values were also determined experimentally in the whole concentration range via the

pendant drop method (see section 1.5) and the results are presented in Table S1. The adopted approach was comprised of the following steps: *(i)* The difference of the cosines between θ at each applied potential and θ_{pzc}, $\Delta cos\theta$, was calculated by inserting in the Y-L equation the corresponding C, γ_{LV} and E_{pzc} values for the whole c_{KF} range studied (blue squares in Figure 2). *(ii)* From the C vs. E plots in Figure S4, it is seen that the variations of the capacitance with applied potential with respect to the capacitance at E_{pzc} for each c_{KF} and within the purely capacitive window are less than 2.5 μF cm^{-2} in line with similar studies reported in the literature using aqueous NaF solutions for electrolyte concentrations ranging from 10^{-5} to 0.9 M [3,4]. These relatively small differences render the effect of C on $\Delta cos\theta$ less significant than that of the quadratic potential term in the Y-L equation. On this basis, we predicted the Y-L-type curve (red lines in Figure 2) in the whole potential window studied, by fitting a parabolic function of the form $y = a(x - c)^2$ in the estimated electrowetting data from step *(i)*. In this sense, the parameter a in the fitted equation can be considered as a weighted average value of the capacitance measurements within the capacitive region (effective capacitance).

Table S1. Liquid-air surface tension, γ_{LV}, mass density, ρ, and pH values for KF and CsF solutions in the concentrations used for the electrowetting experiments determined by applying the pendant drop method (see section 1.5). The droplet volume for the surface tension measurements was ca. 15 ± 0.5 μL.

c_{KF} / m	γ_{LV}^{KF} / mN m^{-1}	ρ_{KF}/ g cm^{-3}	pH_{KF}	c_{CsF} / m	γ_{LV}^{CsF} / mN m^{-1}	ρ_{CsF}/ g cm^{-3}	pH_{CsF}
0.1	70.89 (±0.18)	1.015	6.5	0.1	70.49 (±0.06)	1.021	6.5
0.5	72.69 (±0.14)	1.030	6.9	0.5	72.05 (±0.15)	1.076	7.0
1	73.05 (±0.18)	1.052	7.2	1	76.52 (±0.09)	1.126	7.9
5	81.02 (±0.14)	1.216	8.3	5	82.72 (±0.13)	1.619	8.4
10	87.69 (±0.19)	1.291	9.4	10	98.70 (±0.19)	2.156	9.7
16	103.16 (±0.26)	1.498	10.6	20	118.66 (±0.33)	2.842	10.8

2.4 Investigation of the physicochemical processes occurring at the graphite – aqueous KF interface

In Figure S5a the CVs for the 10 and 16 m KF solutions between 0 and – 2 V vs. $Ag/AgCl_{(sat.KCl)}$ are displayed. It can be seen that a reductive charge transfer reaction at ca. – 1.63 V is identified, with its anodic counterpart at ca. – 1.5 V. We assign these processes to the specific adsorption of K^+ ions on the surface of HOPG upon negative potential scan sweep, with their subsequent desorption when the direction of the potential scan is reversed. The use of highly concentrated solutions (c_{KF} > 5 m) renders the detection of such processes feasible because: *(i)* the onset potential of HER is significantly shifted to more negative values compared to the lower electrolyte concentration and *(ii)* the decrease in water to electrolyte molar ratio results in a decrease of the K^+ ions' solvation shell thickness which allows for stronger interactions between the K^+ ions and graphite. The peak separation is determined to be ca. 130 mV, suggesting that the adsorption/desorption processes exhibit sluggish kinetics that might be related to ion-ion interactions and/or surface-confined phenomena [15]. A similar finding has been recently reported by Yasuda et al [15] on Au(111) supported graphene in KOH aqueous solutions in the concentration range between 0.01 to 1 M. In the same work, the peak separation at a scan rate of 1 V s^{-1} is found to be ca. 200 mV.

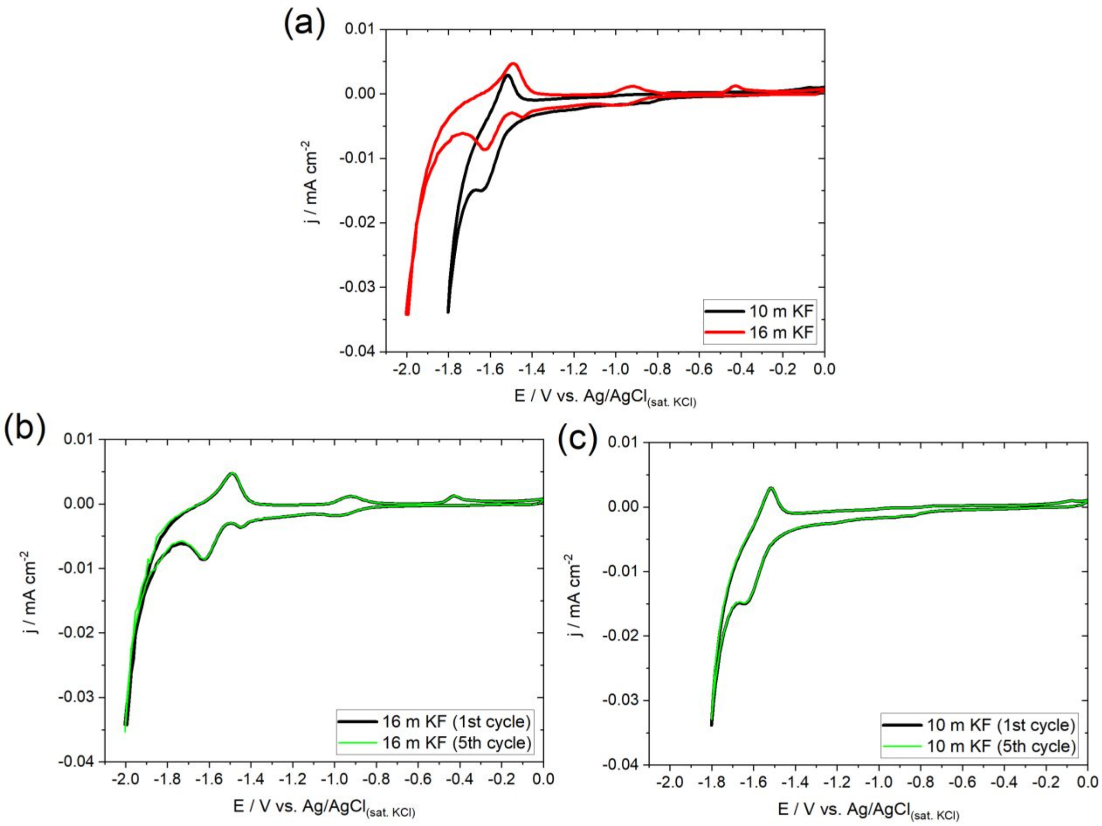

Figure S5. (a) Cyclic voltammograms recorded on freshly cleaved HOPG electrodes using the Teflon cell setup (see Figure S1b and section 1.3 in the SI) in the potential range between 0 up to – 2 V vs. Ag/AgCl$_{(sat. KCl)}$ at a scan rate of 1 V s^{-1} in KF solutions of 10 and 16 m. (b-c) Same as in (a) including the fifth stabilized potential cycle.

Figures S6a-b show the current density dependence at +1.8 V vs. vs. Ag/AgCl $_{(sat.KCl)}$ on scan rate, v, for the 10 m KF solution (Figure 4a). As discussed in the main text, the linear relation between j and $v^{1/2}$ suggests a diffusion-controlled reaction in which F^- ions are actively involved. Groult et al. [16] studied the effect of water content on the fluorine evolution reaction (FER) in molten KF – 2HF on various types of carbon anodes, including HOPG. They concluded that FER is accelerated in solutions of decreased water content and increased F^- concentration. In the presence of water, the reaction proceeds simultaneously with OER and the formation of CO, CO_2, COF_2 and F_2O. In the same study, the formation of a solid carbon-fluoride film on the carbon anodes during electrolysis was confirmed by XPS and scanning tunneling microscopy. This film is composed of conducting fluorine – graphite intercalation compounds (GIC) and a small amount of insulating graphite fluorides. The latter increases with an increase in water content and the rate of FER is significantly decreased.

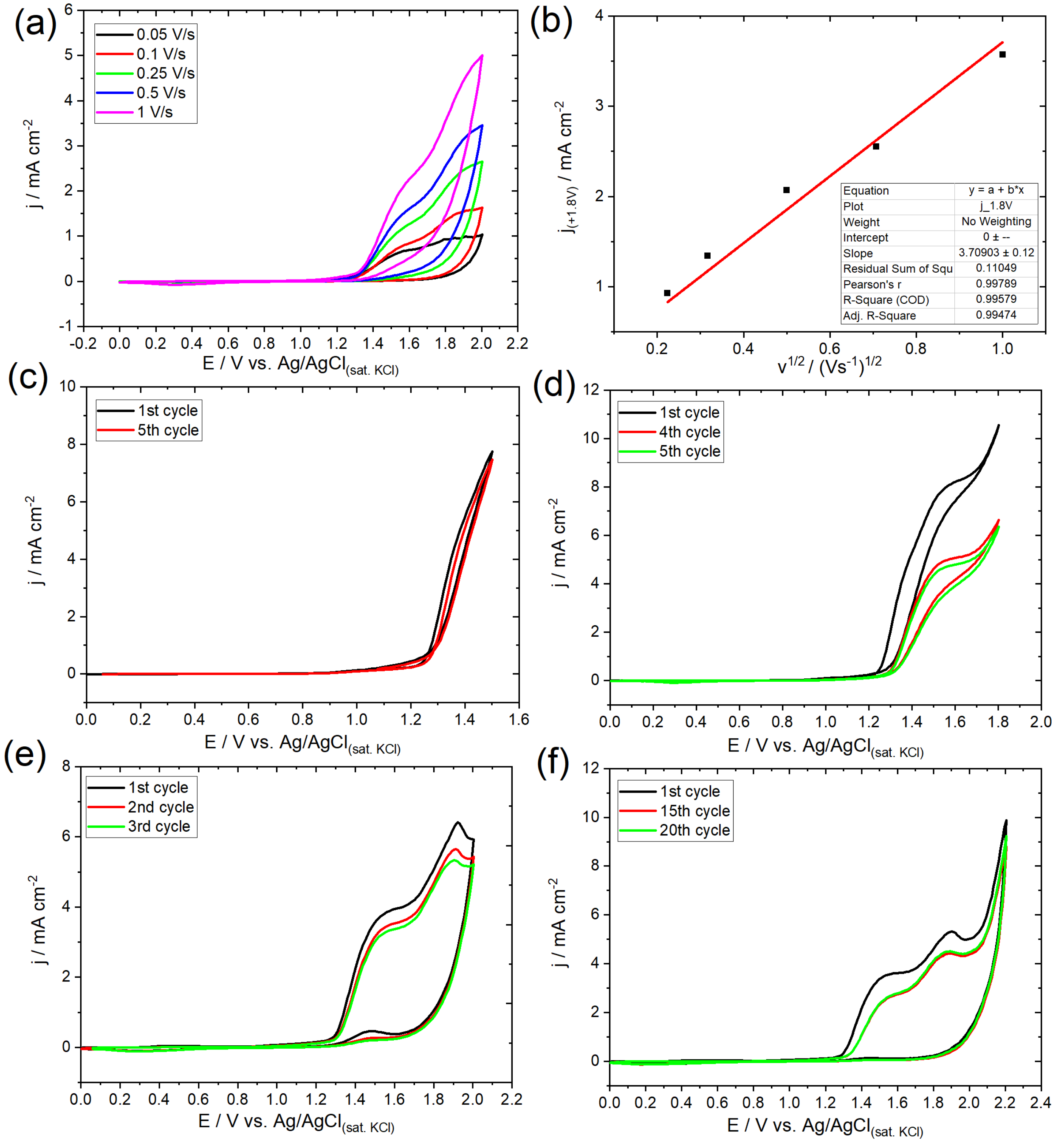

Figure S6. (a) Cyclic voltammograms recorded on freshly cleaved HOPG electrodes using the Teflon cell setup (see Figure1b and Methods) in the potential range between 0 to +2 V vs. $Ag/AgCl_{(sat.\ KCl)}$ at various scan rates, υ, in 10 m KF solutions. (b) The dependence of current density, j, (per electrode nominal area) determined at +1.8 V vs. $Ag/AgCl_{(sat.KCl)}$ on the square root of scan rate. Data obtained from (a). (c-f) Same as in (a) for multiple cycles and varying anodic potential limits.

To investigate the possibility of the occurrence of similar type of processes in our system, we performed XPS measurements on anodically treated HOPG electrodes in 10 m KF (Figure 4b and Figure S7). The treatment procedure was comprised of two steps: *(i)* a potential pulse at +1.8 V vs. $Ag/AgCl_{(sat.KCl)}$ for 1000 s and *(ii)* rinsing of the sample with copious amounts of water to remove any residual electrolyte prior to the

XPS experiments. The applied bias during step *(i)* was based on the features of the CV presented in Figure 4a and it was chosen such that it corresponds to a sufficiently positive value within the potential region where FER related reactions might occur. For comparison purposes we also prepared an HOPG sample on which a droplet of 10 m KF was deposited and subsequently left to evaporate in ambient conditions. Then the area under the droplet was thoroughly rinsed with water to remove any salt residue. Following the discussion in the main text, Figure S7b presents the high-resolution spectra of the K2p region, where the absence of a substantial peak assignable to potassium confirms the formation of semi-ionic fluorine – graphite intercalation compounds. A C-F environment in C1s signal is not observed due to the presence of the high HOPG substrate signal, which masks the relatively minor C-F contribution. Based on these findings, we conclude that the observed anodic oxidation wave is attributed to the formation of FER intermediates (similar to those detected by Groult et al. [16]) most probably in the form of fluorine – graphite intercalation compounds at a relatively low degree of surface coverage. The surface coverage was estimated using the XPS data to be ca. 6% and 46% of a monolayer for the covalently bonded and the semi-ionic fluorine, respectively. However, to provide more accurate insights into the nature and degree of surface coverage of these surface films, additional spectroscopic studies are needed. It is though noteworthy that we detected the presence of such GIC films at a significantly lower potential (+1.8 V vs. $Ag/AgCl_{(sat.KCl)}$) compared to that reported by Groult et al. [16] (> +3.2 V vs. Cu/CuF_2 or ca. +3.28 V vs. $Ag/AgCl_{(sat.KCl)}$ [17]). The conductivity of the film formed was confirmed by monitoring the dependence of current density on the number of potential scan cycles. Within the potential range where electrowetting was investigated (Figure S6c) no dependence of the current density on the number of potential cycles is observed, which demonstrates the conducting character of the electrode (as can be also inferred by the dynamic measurements presented in Figure 7). Upon gradually expanding the anodic potential limit (Figures S6d-f) towards the onset of electrolyte decomposition, a decrease in the current density is observed during the initial cycles (being more evident in Figure S6d). However, the voltammetric response is stabilized after further cycling and still delivers high currents (in the order of several mA cm^{-2}). Following the preceding discussion, the initial decrease in current density seen might be ascribed to the generation of gaseous FER intermediate products that block a fraction of the electrode's active sites (resulting in an overestimation of the electrode's electroactive surface area) and the gradual increase in the surface coverage of covalent-type C-F species.

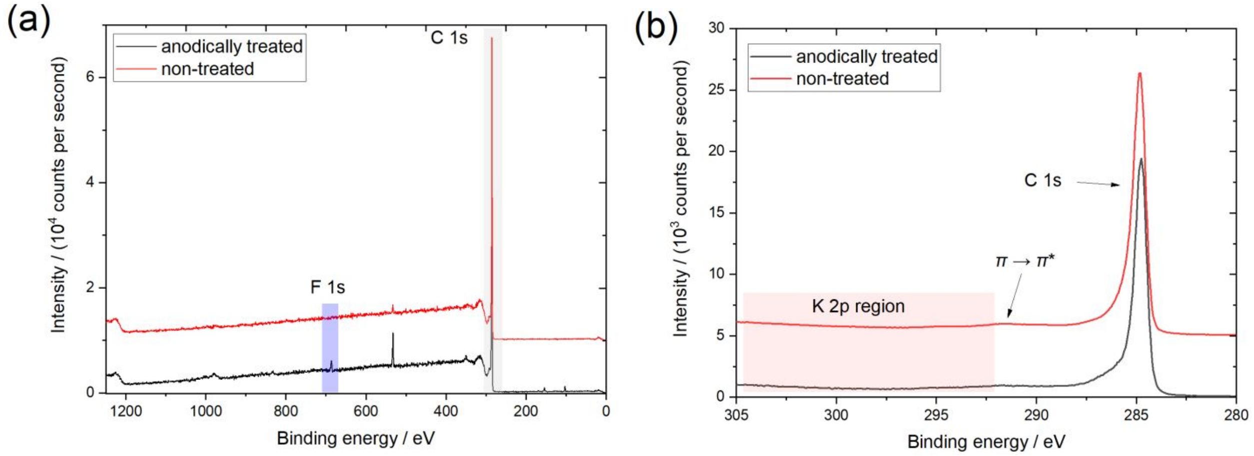

Figure S7. XPS data for the anodically treated and non-treated HOPG samples in 10 m KF; (a) XPS survey spectra of both samples. (b) XPS narrow window scans in the K2p binding energy region.

Figure S8 presents the dependence of θ relative to θ_{pzc} and j at applied potential bias of +1.5 V on c_{KF}. It is shown that j increases almost linearly with C_{KF} up to 10 m and then decreases about 14% for the 16 m solution. This in contrast to what has been recorded for $E < E_{pzc}$ (Figure 3c) and highlights the fact that despite the modification of graphite's surface upon interaction with the electrolyte anions at the positive potential limit of the electrowetting curves (see Figure 4, Figure S6 and the relevant discussion in the main text and SI), the electrowetting response is unaffected. This is a rather interesting finding since it is well

established in the literature that functionalization of graphite with fluorine increases its hydrophobic character [18,19]. However, in our case the main product of the anodic reaction in the concentrated electrolytes within the potential window used, is - as confirmed by the XPS measurements (see Figure 4b in the main text and the relevant discussion therein) - the formation of fluorine GIC compounds with ionic and/or semi-ionic bonds, a process referred in the literature as soft fluorination[16]. In contrast with fluorinated carbon films [18,19], the formation of GIC layers seems not to inhibit the hydrophilic character of graphite. Finally, the apparent decrease of j for the 16 m electrolyte, may be attributed to the increase in the viscosity of the solution at this highly concentrated regime [1] that is expected to decrease the diffusion coefficient of the F^- ions and thus affect the rate of the diffusion controlled process (see Figure S6). At the same time, increased repulsive interactions among F^- ions on the surface of graphite can partially hinder the formation of the semi-ionic GIC surface film, resulting to a decrease in the overall rate of the process.

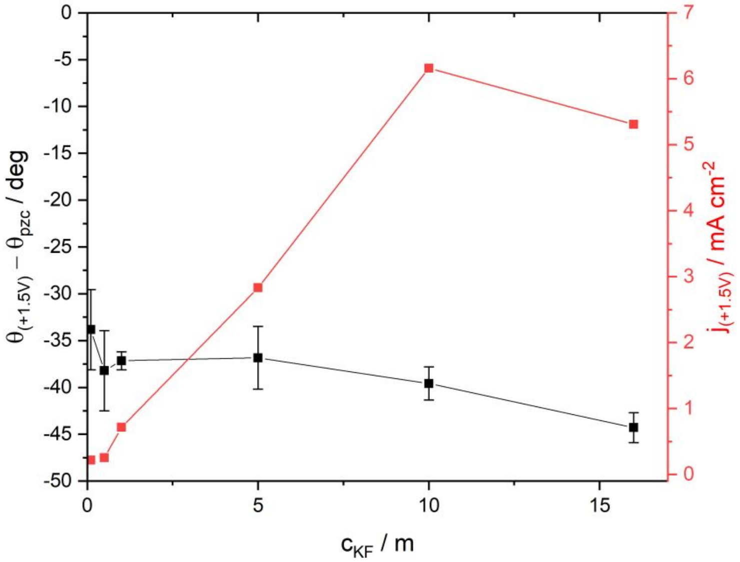

Figure S8. Apparent equilibrium electrowetting contact angle, θ, relative to θ_{pzc} and current density, j, for each c_{KF} at applied potential +1.5 V ($\theta_{(+1.5V)}$ and $j_{(+1.5V)}$) vs. Ag/AgCl wire and Ag/AgCl$_{(sat.\ KCl)}$, respectively. The applied potential corresponds to the most positive potential applied in the electrowetting curves of Figure 1b.

2.5 The effect of electrolyte identity on the electrowetting curves

Figure S9 shows the dependence of the apparent contact angle, θ, on the applied potential, E, for c_{CsF} in the range of 0.5 to 20 m. The standard deviations for all measurements are also included. The data highlights the negative effect of decreasing c_{CsF} on the electrowetting response, in line with what is reported for the KF solutions (see Figure 1a). Furthermore, in Figure S10, the Bode plots recorded in the high concentration regime for CsF, i.e., 10 and 20 m are presented. For the 20 m solution, the purely capacitive window is determined to be 2.8 V highlighting the beneficial effect of using highly concentrated solutions on the electrolyte stability window and thus the electrowetting response of the system (see Figure 1-2 and Figure 6 as well as the relevant discussion in the main text).

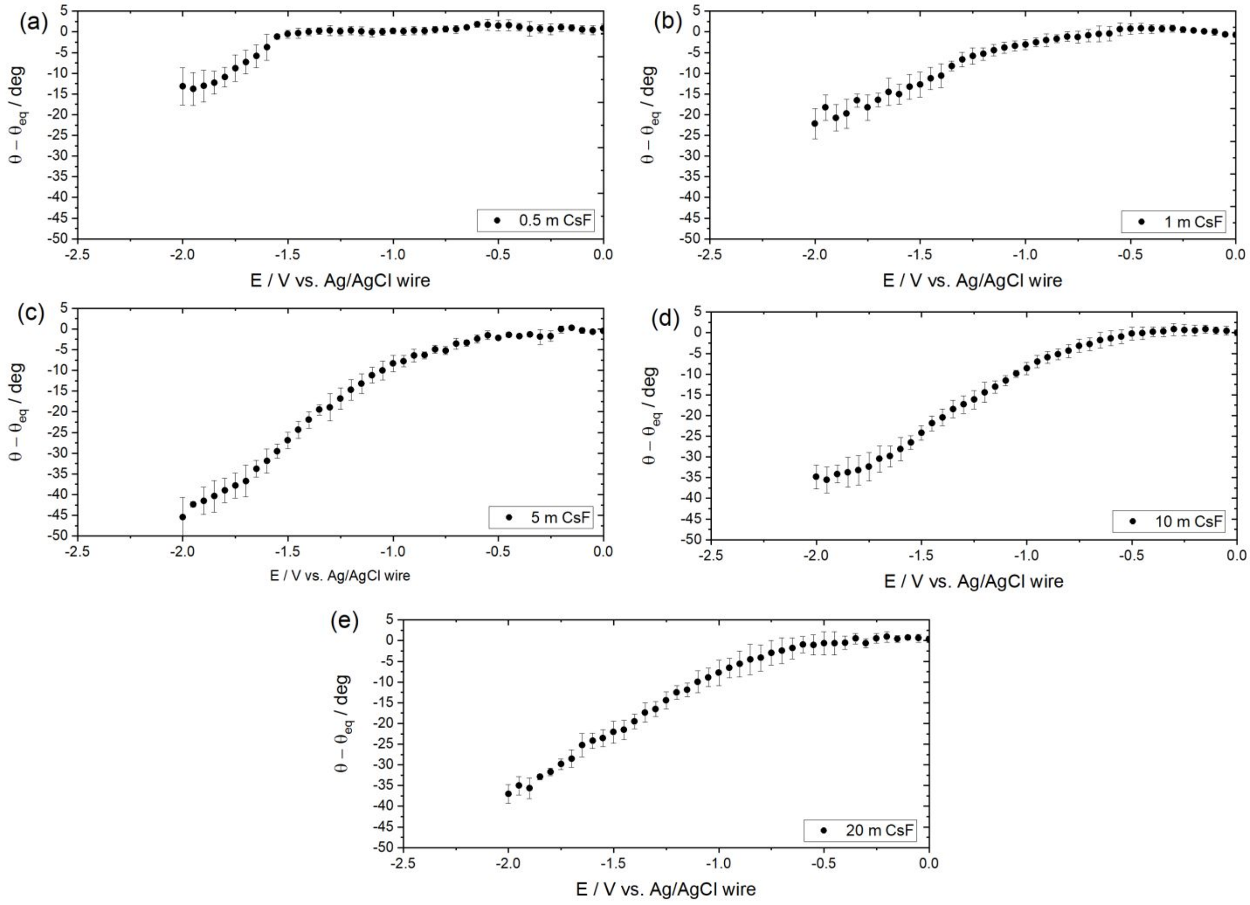

Figure S9. Changes in apparent contact angle, θ, as recorded in the potential range within 0 and -2 V vs. Ag/AgCl wire between (a) 0.5 m, (b) 1 m, (c) 5 m CsF, (d) 10 m CsF and (e) 20 m CsF solutions. The results correspond to averages and standard deviations of no less than five experiments on freshly cleaved HOPG. Measurements were conducted under static conditions based on the protocol described in Methods.

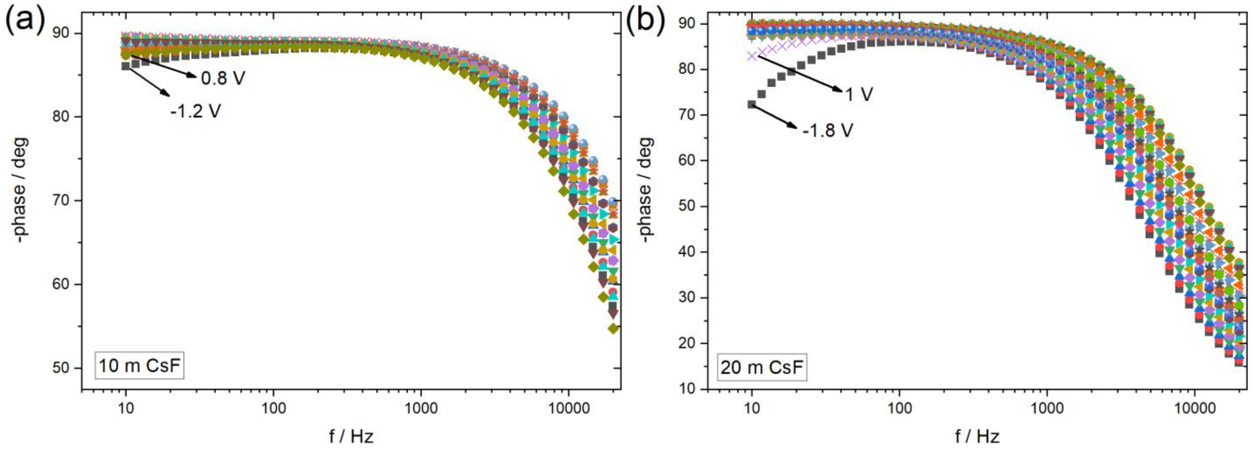

Figure S10. EIS spectra adopting Bode representation in (a) 10 m and (b) 20 m CsF solutions. Experiments were conducted in the frequency range between 20 kHz and 10 Hz with an imposed AC rms amplitude of 7 mV peak-to-peak. The experiments were performed using the Teflon cell setup (see Figure 1b and section 1.3 herein) on freshly cleaved HOPG samples.

Finally, the effect of the electrolyte anion on the electrowetting response for applied bias more positive than the E_{pzc} was also investigated and the results are presented in Figure S11. It can be seen that the identity of the electrolyte seems to have no significant effect on the electrowetting response for $E > E_{pzc}$.

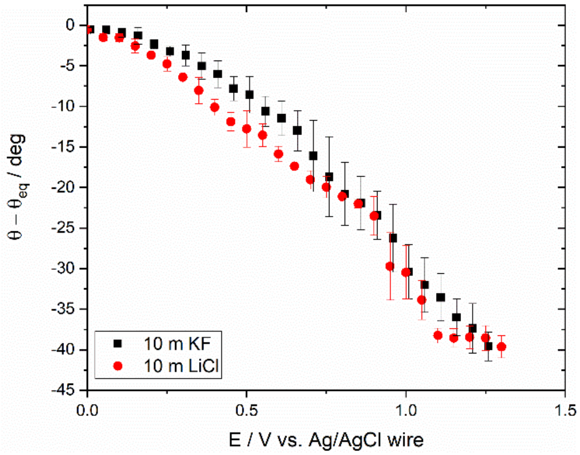

Figure S11. Comparison of the change in apparent contact angle, θ, as recorded in the potential range within 0 and 1.25 V vs. Ag/AgCl wire between 10 m KF and LiCl solutions. The results correspond to averages and standard deviations of no less than five experiments on freshly cleaved HOPG. Measurements were conducted under static conditions based on the protocol described in Methods.

2.6 Dynamic measurements

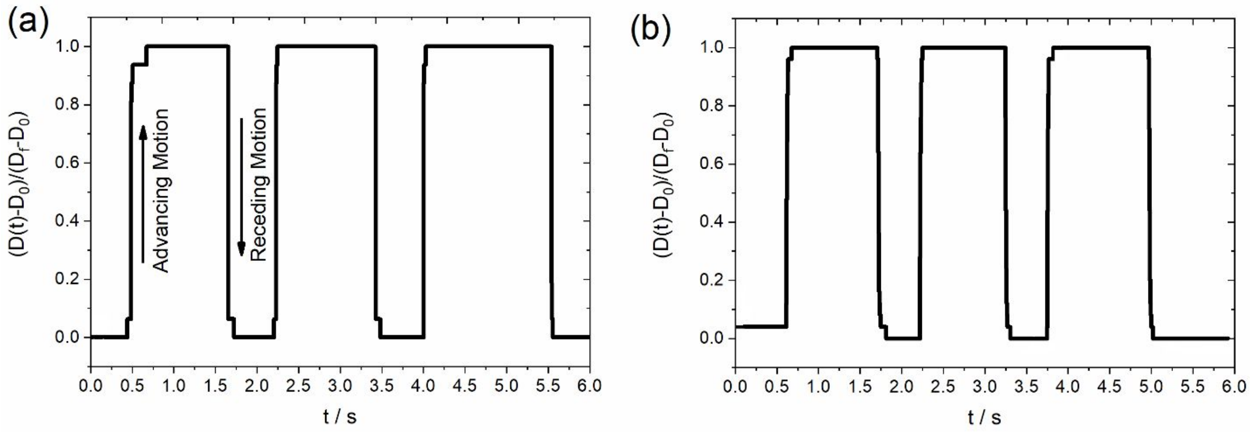

Figure S12. Change in droplet diameter, d, during wetting/dewetting cycles for the data presented in Figure 5. One cycle corresponds to two consecutive potential pulses from 0 to (a) -2 V (maximum change in d for $E < E_{pzc}$) and (b) +1.5 V (maximum change in d for $E > E_{pzc}$) vs. Ag/AgCl wire. D_0 and D_f are the equilibrium and maximum droplet diameter respectively, while $D(t)$ is the diameter value for each t.

The changes in droplet diameter during the consecutive wetting/dewetting cycles of the data presented in Figure 7, are shown in Figure S12. The results demonstrate the high degree of reversibility among the advancing and receding motions of the droplet, that in line with the CA changes presented in Figure 7 prove the high reproducibility of the electrowetting process.

The dependence of the specific conductance of the various KF aqueous solutions, σ_{KF}, on electrolyte concentration is depicted in Figure S13. Within the low concentration regime, i.e., c_{KF} < 5 m, the data shows a linear increase of σ_{KF} with an increase in c_{KF}. For higher electrolyte concentrations, the recorded trend exhibits a nonlinear increase up to a maximum value -obtained for 10 m- and then it decreases on a further increase of the electrolyte concentration. This finding is in line with similar measurements reported in the literature for alkali halides electrolytes [20,21] and LiTFSI (lithium bis(trifluoromethanesulfonyl)imide) [22] up to the water-in-salt concentration regime. The decrease in electrolyte conductivity observed in the highly concentrated solutions (here c_{KF} > 10 m) can be ascribed to the increased amount of electroneutral species in solution as a consequence of the higher degree of ionic association (ion pairing occurs) as well as the increase in relaxation forces and electrophoretic effects that retard the drift of the ions in the solution [20].

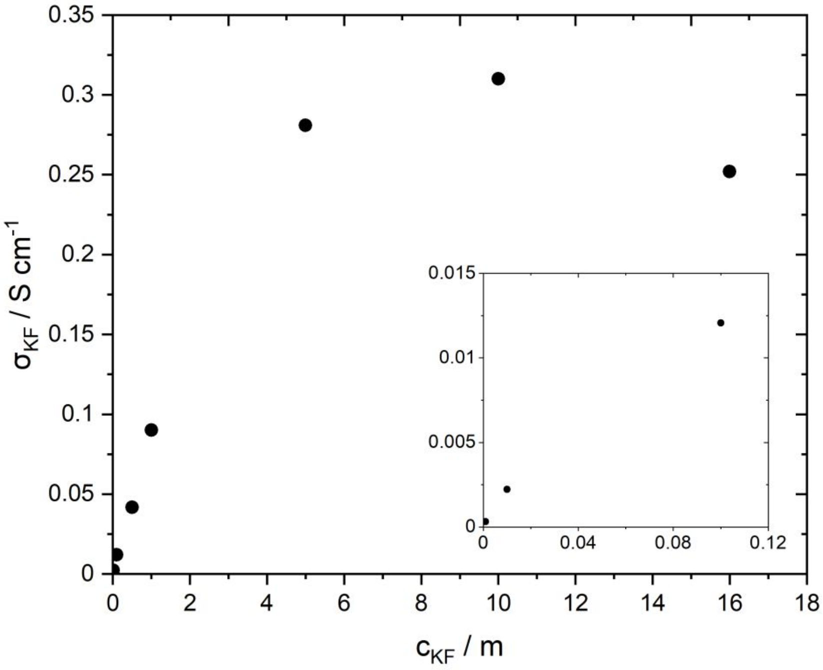

Figure S13. Changes in electrolyte specific conductance with respect to c_{KF} as obtained experimentally using an electronic conductivity meter (see section 1.5). Inset: Magnification of the low c_{KF} regime.

2.7 Optical images of the droplets at selective potential biases for various electrolytes

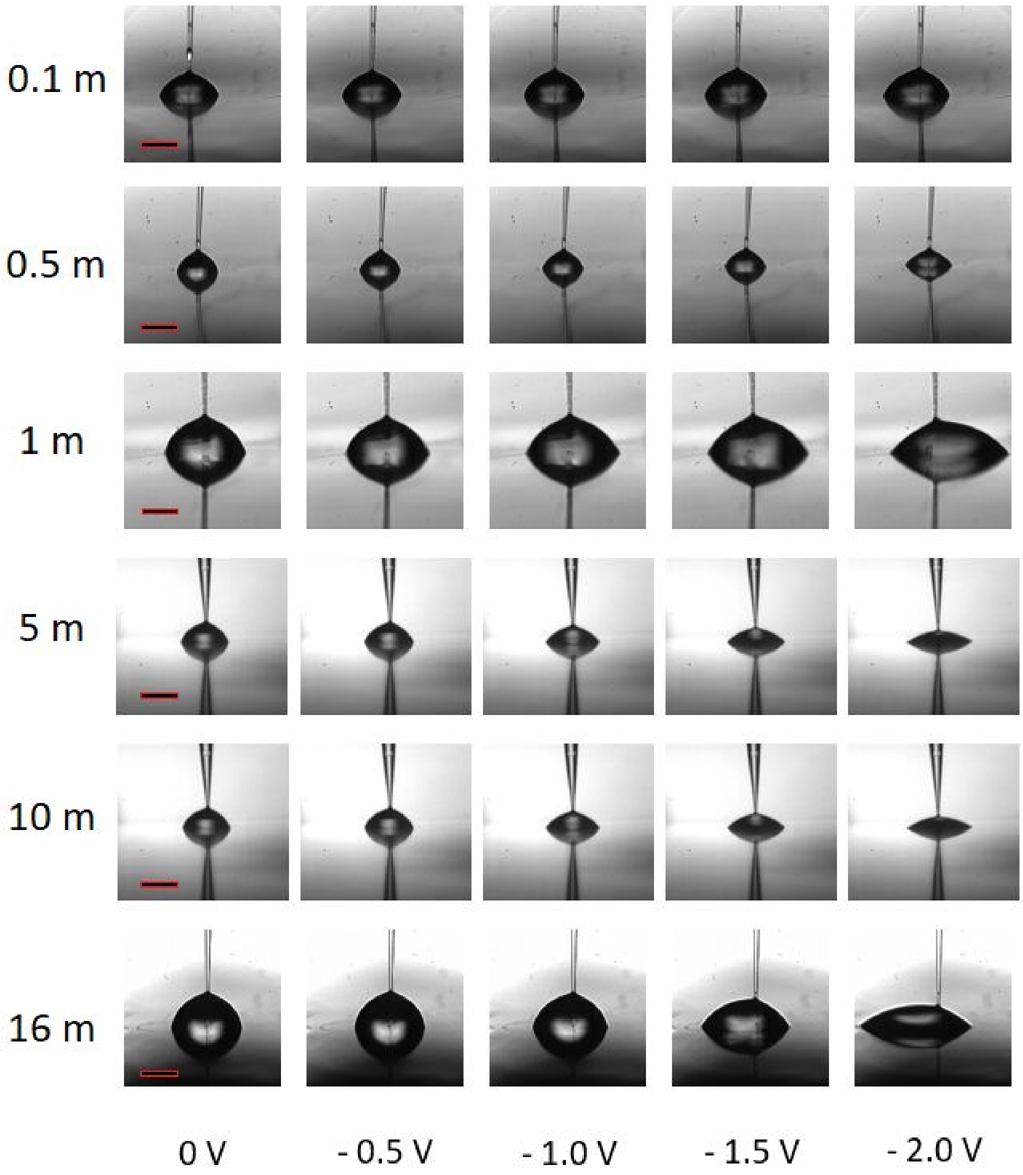

Figure S14. Droplet pictures at indicative potentials within the negative potential window of the results presented in Figure 1c for various KF concentrations. Potentials are quoted vs. Ag/AgCl wire. Scalebars correspond to ca. 100 µm.

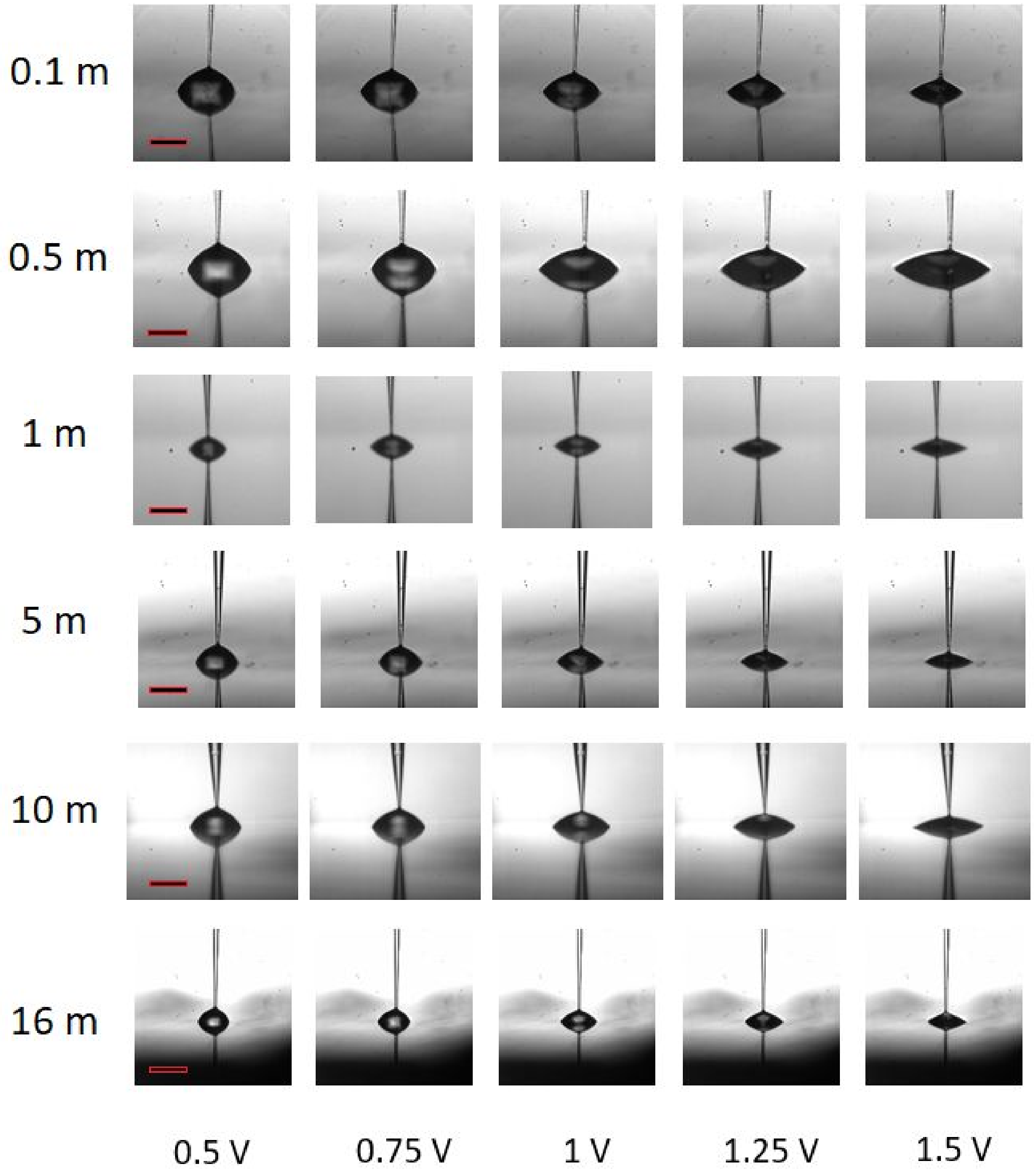

Figure S15. Droplet pictures at indicative potentials within the positive potential window of the results presented in Figure 1c for various KF concentrations. Potentials are quoted vs. Ag/AgCl wire. Scalebars correspond to ca. 100 μm.

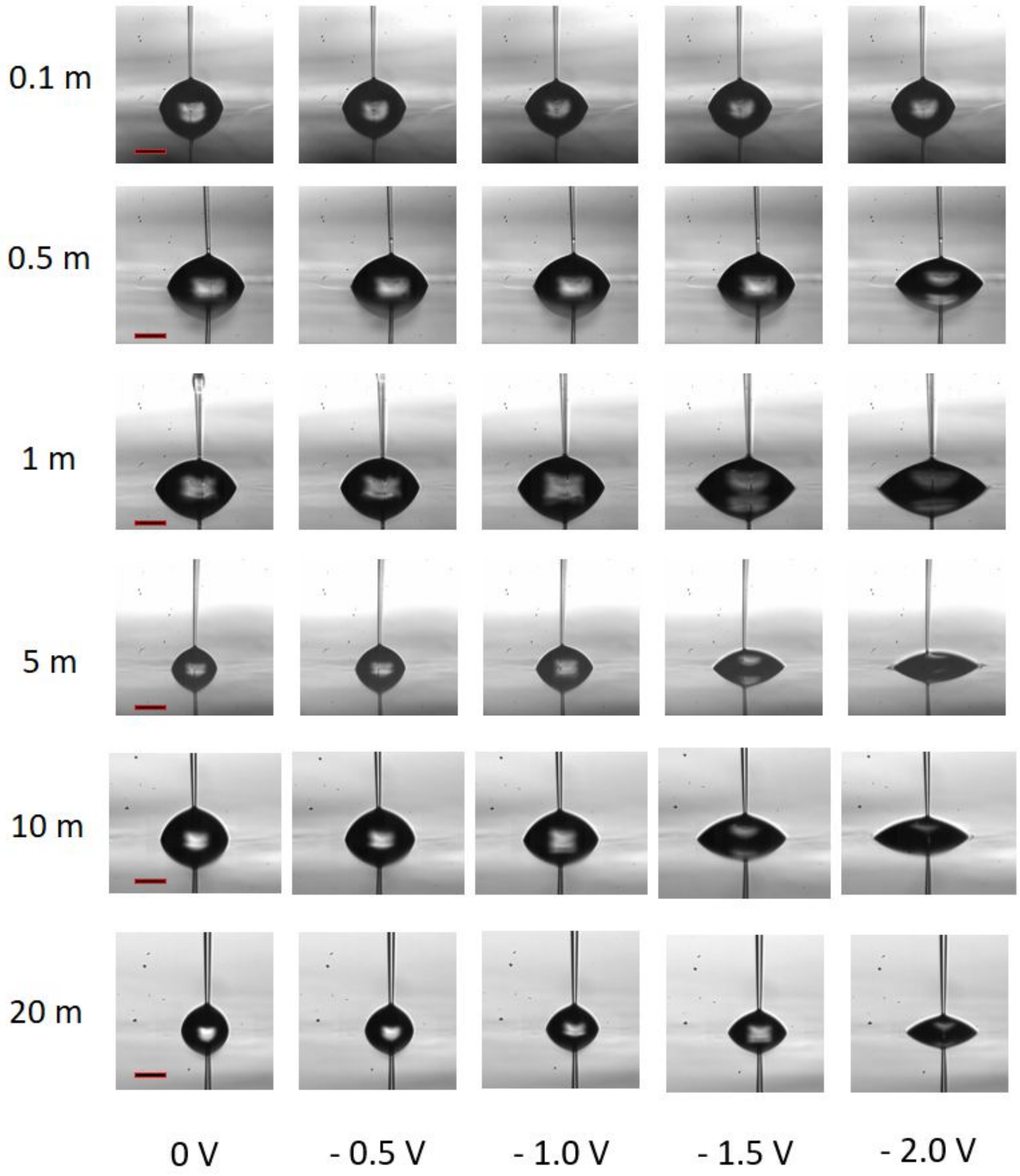

Figure S16. Droplet pictures at indicative potentials within the negative potential window of the results presented in Figure 1d for various CsF concentrations. Potentials are quoted vs. Ag/AgCl wire. Scalebars correspond to ca. 100 μm.

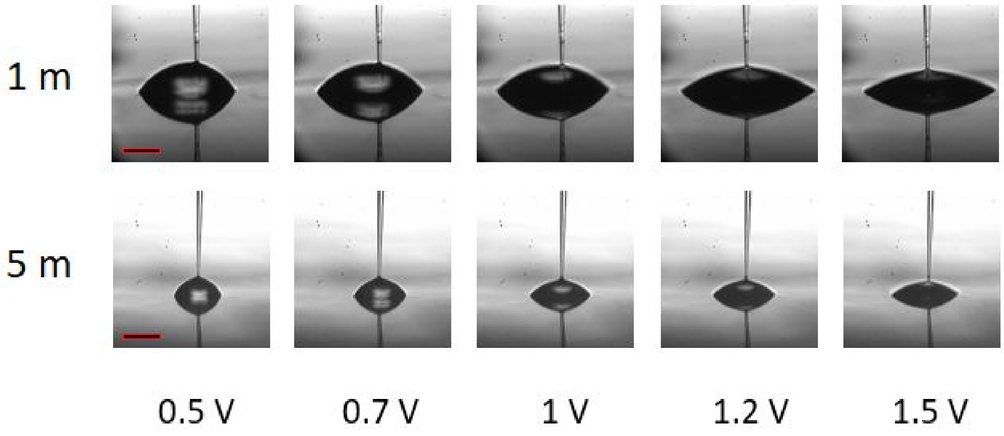

Figure S17. Droplet pictures at indicative potentials within the positive potential window of the results presented in Figure 1d for 1 and 5 m CsF concentrations. Potentials are quoted vs. Ag/AgCl wire. Scalebars correspond to ca. 100 μm.

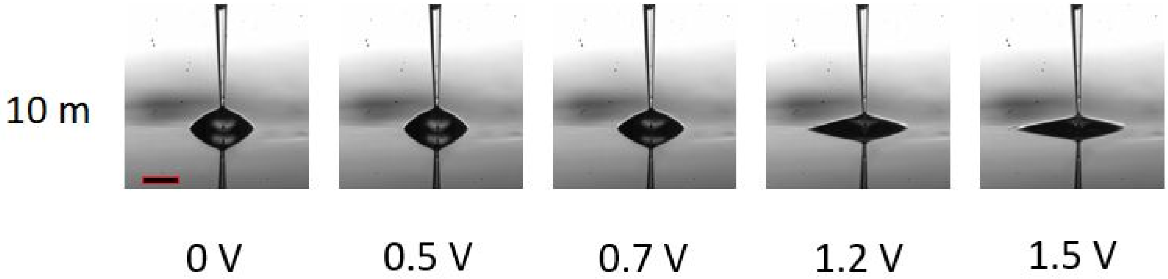

Figure S18. Droplet pictures at indicative potentials within the positive potential window of the results presented in Figure 11 for 10 m LiCl concentrations. Potentials are quoted vs. Ag/AgCl wire. Scalebar correspond to ca. 100 μm.

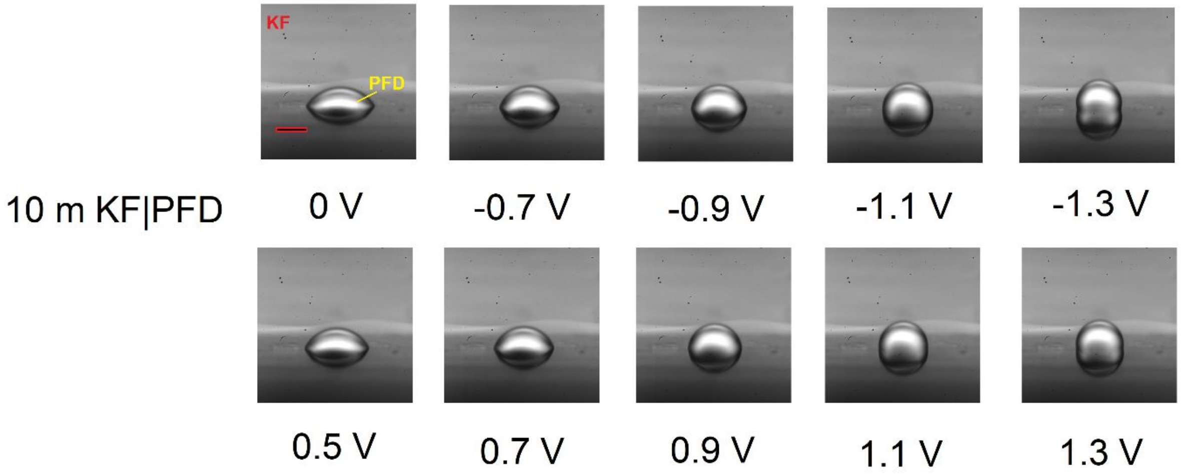

Figure S19. Droplet pictures at indicative potentials within the positive potential window of the results presented in Figure 8a-b for the 10 m KF|PFD liquid|liquid interface. Potentials are quoted vs. Ag/AgCl$_{(sat.\ KCl)}$. Scalebar correspond to ca. 100 µm.

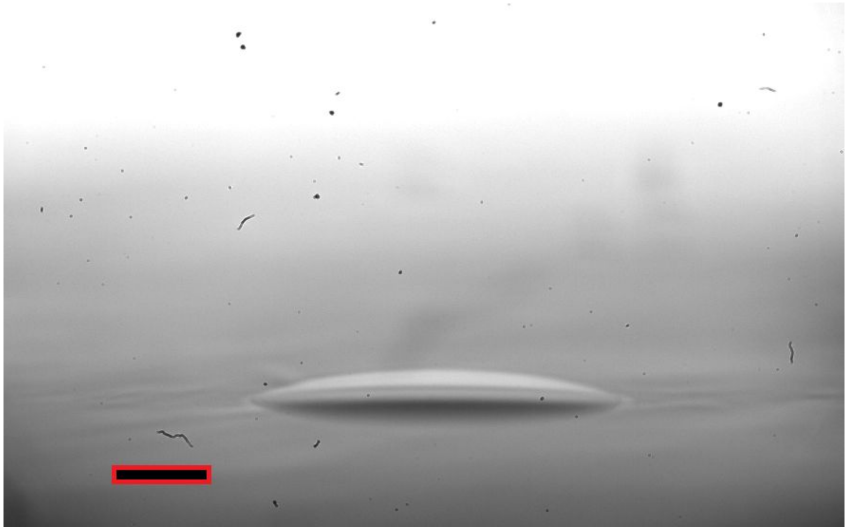

Figure S20. Droplet picture of a PFD droplet in air. Scalebar correspond to ca. 100 µm.

2.8 Captions of movie files

Movie 1: Changes in apparent contact angle, θ, during 200 wetting/dewetting cycles following the protocol described in Methods, for a 10 m KF droplet on HOPG in air (see Figure 6a). One cycle corresponds to two consecutive potential pulses from 0 to -1.6 V vs. Ag/AgCl wire. The playback speed is set to 125 fps, i.e., five times faster than that in the actual recording (50 fps).

Movie 2: Changes in apparent contact angle, θ, during 200 wetting/dewetting cycles following the protocol described in Methods, for a 10 m KF droplet on HOPG in air (see Figure 6b). One cycle corresponds to two consecutive potential pulses from 0 to +1 V vs. Ag/AgCl wire. The playback speed is set to 125 fps, i.e., five times faster than that in the actual recording (50 fps).

Movie 3: Changes in apparent contact angle, θ, during 200 wetting/dewetting cycles following the protocol described in Methods, at the PFD|10 m $KF_{(aq)}$ liquid|liquid interface (see Figure 8c). One cycle corresponds to two consecutive potential pulses from 0 to -1.6 V vs. $Ag/AgCl_{(sat.\ KCl)}$. The playback speed is set to 125 fps, i.e., five times faster than that in the actual recording (50 fps).

Movie 4: Changes in apparent contact angle, θ, during 200 wetting/dewetting cycles following the protocol described in Methods, at the PFD|10 m $KF_{(aq)}$ liquid|liquid interface (see Figure 8d). One cycle corresponds to two consecutive potential pulses from 0 to +1 V vs. $Ag/AgCl_{(sat.\ KCl)}$. The playback speed is set to 125 fps, i.e., five times faster than that in the actual recording (50 fps).

References

(1) Iamprasertkun, P.; Ejigu, A.; Dryfe, R. A. W. Understanding the Electrochemistry of "Water-in-Salt" Electrolytes: Basal Plane Highly Ordered Pyrolytic Graphite as a Model System. *Chem. Sci.* **2020**, *11* (27), 6978–6989. https://doi.org/10.1039/D0SC01754J.

(2) Pletcher, D. *A First Course in Electrode Processes*, 2nd ed.; RSC publishing: Cambridge, UK, 2009.

(3) Randin, J.-P.; Yeager, E. Differential Capacitance Study on the Basal Plane of Stress-Annealed Pyrolytic Graphite. *Journal of Electroanalytical Chemistry and Interfacial Electrochemistry* **1972**, *36* (2), 257–276. https://doi.org/10.1016/S0022-0728(72)80249-3.

(4) Randin, J.-P.; Yeager, E. Differential Capacitance Study of Stress-Annealed Pyrolytic Graphite Electrodes. *J. Electrochem. Soc.* **1971**, *118* (5), 711. https://doi.org/10.1149/1.2408151.

(5) Gerischer, H.; McIntyre, R.; Scherson, D.; Storck, W. Density of the Electronic States of Graphite: Derivation from Differential Capacitance Measurements. *J. Phys. Chem.* **1987**, *91* (7), 1930–1935. https://doi.org/10.1021/j100291a049.

(6) Mile, V.; Gereben, O.; Kohara, S.; Pusztai, L. On the Structure of Aqueous Cesium Fluoride and Cesium Iodide Solutions: Diffraction Experiments, Molecular Dynamics Simulations, and Reverse Monte Carlo Modeling. *J. Phys. Chem. B* **2012**, *116* (32), 9758–9767. https://doi.org/10.1021/jp301595m.

(7) Mugele, F.; Heikenfeld, J. Principles of Modern Electrowetting. In *Electrowetting*; John Wiley & Sons, Ltd, 2018; pp 133–157. https://doi.org/10.1002/9783527412396.ch5.

(8) Mugele, F. Fundamental Challenges in Electrowetting: From Equilibrium Shapes to Contact Angle Saturation and Drop Dynamics. *Soft Matter* **2009**, *5* (18), 3377–3384. https://doi.org/10.1039/B904493K.

(9) Iamprasertkun, P.; Hirunpinyopas, W.; Keerthi, A.; Wang, B.; Radha, B.; Bissett, M. A.; Dryfe, R. A. W. Capacitance of Basal Plane and Edge-Oriented Highly Ordered Pyrolytic Graphite: Specific Ion Effects. *J. Phys. Chem. Lett.* **2019**, *10* (3), 617–623. https://doi.org/10.1021/acs.jpclett.8b03523.

(10) Williams, C. D.; Dix, J.; Troisi, A.; Carbone, P. Effective Polarization in Pairwise Potentials at the Graphene–Electrolyte Interface. *J. Phys. Chem. Lett.* **2017**, *8* (3), 703–708. https://doi.org/10.1021/acs.jpclett.6b02783.

(11) Zhan, C.; Cerón, M. R.; Hawks, S. A.; Otani, M.; Wood, B. C.; Pham, T. A.; Stadermann, M.; Campbell, P. G. Specific Ion Effects at Graphitic Interfaces. *Nature Communications* **2019**, *10* (1), 4858. https://doi.org/10.1038/s41467-019-12854-7.

(12) Brown, M. A.; Goel, A.; Abbas, Z. Effect of Electrolyte Concentration on the Stern Layer Thickness at a Charged Interface. *Angewandte Chemie* **2016**, *128* (11), 3854–3858. https://doi.org/10.1002/ange.201512025.

(13) Gerischer, H. An Interpretation of the Double Layer Capacity of Graphite Electrodes in Relation to the Density of States at the Fermi Level. *J. Phys. Chem.* **1985**, *89* (20), 4249–4251. https://doi.org/10.1021/j100266a020.

(14) Arscott, S. Moving Liquids with Light: Photoelectrowetting on Semiconductors. *Sci Rep* **2011**, *1* (1), 184. https://doi.org/10.1038/srep00184.

(15) Yasuda, S.; Tamura, K.; Kato, M.; Asaoka, H.; Yagi, I. Electrochemically Driven Specific Alkaline Metal Cation Adsorption on a Graphene Interface. *J. Phys. Chem. C* **2021**, *125* (40), 22154–22162. https://doi.org/10.1021/acs.jpcc.1c03322.

(16) Groult, H.; Durand-Vidal, S.; Devilliers, D.; Lantelme, F. Kinetics of Fluorine Evolution Reaction on Carbon Anodes: Influence of the Surface C-F Films. *Journal of Fluorine Chemistry* **2001**, *107* (2), 247–254. https://doi.org/10.1016/S0022-1139(00)00366-3.

(17) Burrows, B.; Jasinski, R. The Cu / CuF_2 Couple in Anhydrous Hydrogen Fluoride. *J. Electrochem. Soc.* **1968**, *115* (4), 348. https://doi.org/10.1149/1.2411189.

(18) Chronopoulos, D. D.; Bakandritsos, A.; Pykal, M.; Zbořil, R.; Otyepka, M. Chemistry, Properties, and Applications of Fluorographene. *Applied Materials Today* **2017**, *9*, 60–70. https://doi.org/10.1016/j.apmt.2017.05.004.

(19) Ahmad, Y.; Batisse, N.; Chen, X.; Dubois, M. Preparation and Applications of Fluorinated Graphenes. *C* **2021**, *7* (1), 20. https://doi.org/10.3390/c7010020.

(20) Wu, X.; Gong, Y.; Xu, S.; Yan, Z.; Zhang, X.; Yang, S. Electrical Conductivity of Lithium Chloride, Lithium Bromide, and Lithium Iodide Electrolytes in Methanol, Water, and Their Binary Mixtures. *J. Chem. Eng. Data* **2019**, *64* (10), 4319–4329. https://doi.org/10.1021/acs.jced.9b00405.
(21) Malchik, F.; Shpigel, N.; Levi, M. D.; Mathis, T. S.; Mor, A.; Gogotsi, Y.; Aurbach, D. Superfast High-Energy Storage Hybrid Device Composed of MXene and Chevrel-Phase Electrodes Operated in Saturated LiCl Electrolyte Solution. *J. Mater. Chem. A* **2019**, *7* (34), 19761–19773. https://doi.org/10.1039/C9TA08066J.
(22) Suo, L.; Borodin, O.; Gao, T.; Olguin, M.; Ho, J.; Fan, X.; Luo, C.; Wang, C.; Xu, K. “Water-in-Salt” Electrolyte Enables High-Voltage Aqueous Lithium-Ion Chemistries. *Science* **2015**, *350* (6263), 938–943. https://doi.org/10.1126/science.aab1595.
